# Association of *ZNF331* and *WIF1* methylation in peripheral blood leukocytes with the risk and prognosis of gastric cancer

**DOI:** 10.1186/s12885-021-08199-4

**Published:** 2021-05-15

**Authors:** Chuang Nie, Xu Han, Rongrong Wei, Anastasiia Leonteva, Jia Hong, Xinyu Du, Jing Wang, Lin Zhu, Yashuang Zhao, Yingwei Xue, Haibo Zhou, Wenjing Tian

**Affiliations:** 1 Department of Epidemiology, School of Public Health, Harbin Medical University, Harbin, 157 Baojian Road, Harbin, 150081 Heilongjiang Province People’s Republic of China; 2 Department of Gastroenterological Surgery, Third Affiliated Hospital of Harbin Medical University, 150 Haping Road, Harbin, 150081 Heilongjiang Province People’s Republic of China

**Keywords:** Gastric cancer, DNA methylation, Peripheral blood leukocytes, Propensity score

## Abstract

**Background:**

Peripheral blood leukocyte (PBL) DNA methylation may serve as a surrogate marker to evaluate the susceptibility to and prognosis of gastric cancer (GC). In this study, blood-derived DNA methylation levels of two tumour-related genes, namely, *ZNF331* and *WIF1*, and their impacts on the risk and prognosis of GC were evaluated.

**Methods:**

In total, 398 GC cases and 397 controls were recruited for the study. Then, all cases were followed up for 5 years. *ZNF331* and *WIF1* promoter methylation status in PBLs was measured using a methylation-sensitive high-resolution melting method. Logistic and Cox regression models were used to analyse the correlation between gene methylation and the risk and prognosis of GC. Confounders were balanced through propensity score (PS) matching.

**Results:**

High *ZNF331* methylation significantly decreased GC risk after PS adjustment (OR = 0.580, 95% CI: 0.375–0.898, *P* = 0.015), which also presented in males (OR = 0.577, 95% CI: 0.343–0.970, *P* = 0.038). However, *WIF1* methylation was not associated with GC risk. Additionally, significant combined effects between *ZNF331* methylation and the intake of green vegetables and garlic were observed (OR = 0.073, 95% CI: 0.027–0.196, *P* < 0.001 and OR = 0.138, 95% CI: 0.080–0.238, *P* < 0.001, respectively). Furthermore, *ZNF331* and *WIF1* methylation had no impact on the prognosis of GC.

**Conclusion:**

*ZNF331* methylation in PBLs may affect GC risk in combination with the consumption of green vegetables and garlic and may act as a potential biomarker of GC.

**Supplementary Information:**

The online version contains supplementary material available at 10.1186/s12885-021-08199-4.

## Background

Gastric cancer (GC) is an aggressive disease that is the fifth most prevalent malignancy and the third leading cause of cancer-related deaths worldwide [1]. Multiple factors are involved in the development of GC, including genetic, epigenetic and environmental factors [2]. Epigenetics refers to heritable changes in phenotypes that occur without alterations in DNA nucleoside sequences [3]. Abnormal DNA methylation is an extensively studied epigenetic modification that mainly includes two different forms: genome-wide changes and regional variations [4, 5]. Genome-wide methylation changes, known as global DNA hypomethylation, can contribute to carcinogenesis by inducing the formation of repressive chromosomal structures [6]. Regional methylation variations, particularly aberrant hypermethylation in promoter CpG islands, have been relatively more studied and can lead to the silencing of tumour suppressor genes in almost all cancer types [7]. However, losses of DNA methylation at normally methylated CpG islands were virtually the first recognized epigenetic abnormality that can lead to gene activation [5]. DNA methylation changes have emerged as having promising diagnostic, prognostic, and predictive value in cancer [8]. Additionally, epigenetic mechanisms may function as an interface between environmental factors and the genome. Mounting studies have suggested that exposure to environmental and lifestyle factors can affect methylation status and thus promote tumourigenesis [9–11].

Zinc-finger protein 331 (*ZNF331*) belongs to the zinc-finger gene family, encoding a zinc finger protein that contains a Kruppel-associated box domain that plays an essential part in the transcriptional regulation process [12, 13]. Aberrant promoter hypermethylation of *ZNF331* has been demonstrated to epigenetically promote gastric carcinogenesis through the downregulation of its expression [14]. In addition, several other studies in tissue samples supported that *ZNF331* methylation could function as a potential biomarker in gastrointestinal malignancies, particularly for colorectal cancer detection and prognosis prediction [15–17].

Wnt-inhibitory factor-1 (WIF1) is a secreted repressor that can directly bind to various Wnt ligands and inhibit their activities [18, 19]. Promoter hypermethylation of *WIF1*, leading to silencing of its expression and subsequent aberrant activation of the Wnt signalling pathway, was reported to participate in gastric tumourigenesis [20]. In addition, *WIF1* hypermethylation has been revealed to correlate with poor survival in non-small-cell lung cancer [21], oesophageal squamous cell carcinoma [22], and chondrosarcoma [23].

Most of the previous studies have focused on tissue-derived DNA to investigate the association between gene methylation and cancer risks and prognoses. However, as target tissue of interest is often unobtainable, peripheral blood may serve as an ideal surrogate for the exploration of cancer biomarkers due to its non-invasiveness and high accessibility [24]. DNA methylation changes can be observed in most tumours of all organ systems, which indicates that they may arise everywhere in the body, including peripheral blood leukocytes (PBLs) [25]. To date, several studies have demonstrated the impact of blood-derived DNA methylation on the susceptibility to various cancer types, including GC [26–28]. Therefore, this population-based study was conducted to investigate the correlations of environmental exposures, *ZNF331* and *WIF1* methylation in PBLs, and their interactions with the incidence of GC. Furthermore, all GC patients were followed up to measure the prognostic effect of the genes.

## Methods

### Study population

A total of 398 GC patients pathologically diagnosed from 2010 through 2012 were selected as cases. A total of 397 non-cancerous controls were recruited during the same period, including patients with non-digestive diseases and healthy individuals who received health examinations. The source of these subjects and specific inclusion and exclusion criteria have been described before [29]. Every participant provided written informed consent and donated 5 ml of blood samples. The study was approved by the Human Research and Ethics Committee of Harbin Medical University and conducted in accordance with the Declaration of Helsinki.

### Data collection

All subjects completed a face-to-face questionnaire to obtain demographic features, family history of GC, lifestyle, and dietary habits. The questions on food frequency were modified from a previous study on the basis of eating patterns in northern China [30]. The clinicopathological data were obtained from the electronic medical record system. All cases were followed up for 5 years by telephone interview to collect information on the cause of death and date. Overall survival was considered the primary outcome. Finally, 375 GC patients were available for the survival analysis after excluding patients who lacked follow-up data. Among them, 192 patients died, 139 patients survived, and 44 patients were lost to follow-up. Helicobacter infection status was measured by ELISA (IBL, Germany).

### Methylation detection

Genomic DNA was extracted with a QIAamp DNA Blood Mini Kit (Qiagen), and then bisulfite modification was performed using an EpiTect Fast DNA Bisulfite Kit in accordance with the manufacturer’s protocols. DNA purity and concentrations were quantified by spectrophotometric measurement. Then, primer pairs of the two candidate genes were designed, and Additional file 1 (Table S1) shows the primer sequences and detailed information on the reaction conditions. PCR and methylation-sensitive high-resolution melting (MS-HRM) analysis were conducted on a Roche 480 II (Germany). Methylation status was determined using Gene Scanning software (version 2.0).

**Fig. 1:**
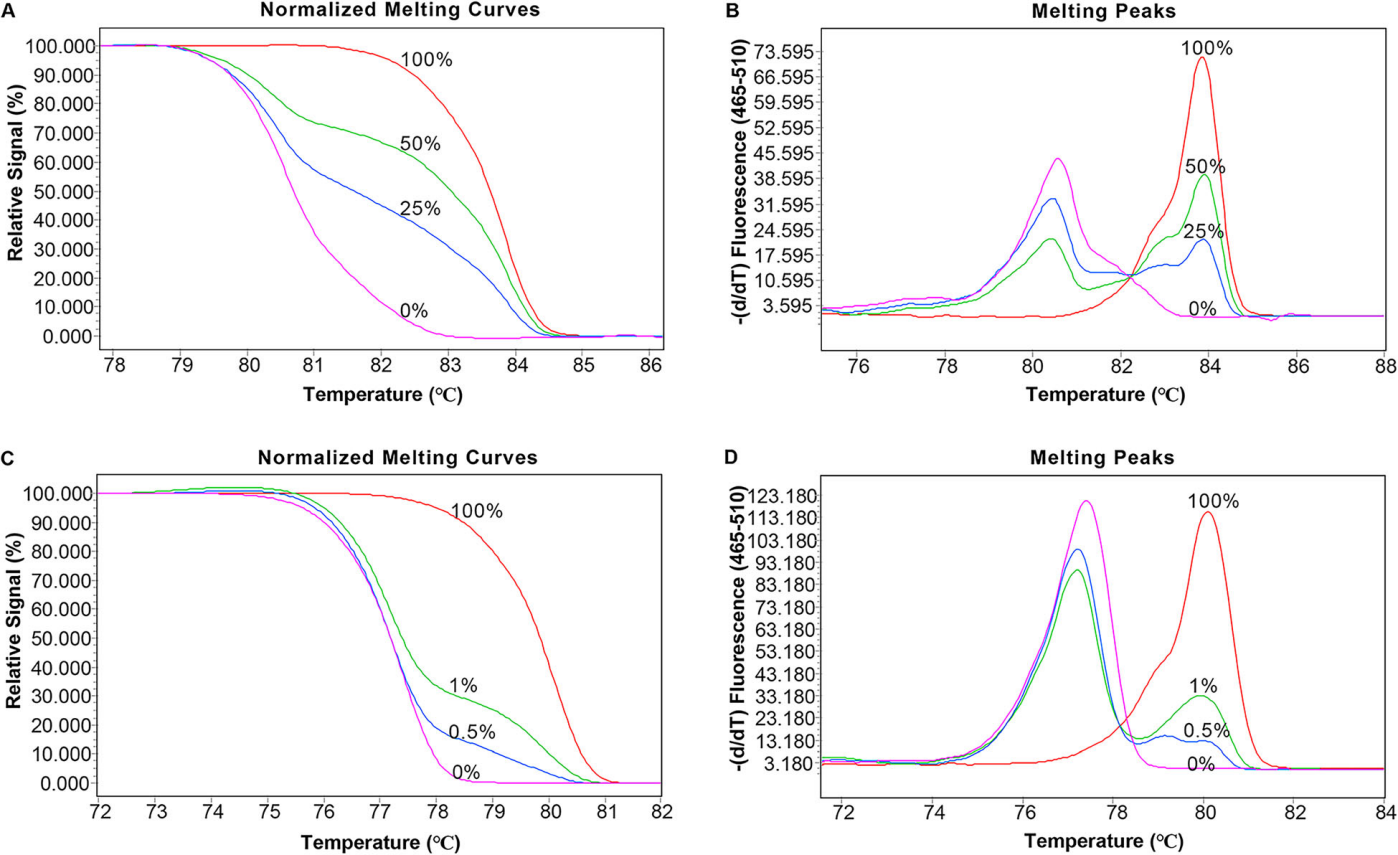
A series of methylated DNA standards were used for *ZNF331* and *WIF1*. **a** Normalized melting curves of the MS-HRM assay for *ZNF331*. **b** *ZNF331* melting peaks were generated by taking the negative derivative (d) of the melting curve data divided by the derivative with respect to time-(d/dT). **c** Normalized melting curves of the MS-HRM assay for *WIF1*. **d** *WIF1* melting peaks were generated by taking the negative derivative (d) of the melting curve data divided by the derivative with respect to time-(d/dT)

A series of normalized curves were constructed by mixing DNA methylated standards (100% methylated and 0% methylated DNA, Zymo Research). The corresponding sequence information of the DNA methylated standards is shown in Additional file 1 (Table S1). PCR-grade water was used as a blank control in every experimental run. Figure 1 presents the standard melting curves and melting peaks. According to the receiver operating characteristic curve, 50% methylated DNA was used as the optimal cut-off value for *ZNF331* to determine high methylation (Hm) or low methylation (Lm) (Additional file 2: Figure S1). For the definition of *WIF1* methylation status, Hm refers to a combination of homogeneous methylation (Hom) and heterogeneous methylation (Hem), and Lm is consistent with 0% methylated DNA (Additional file 3: Figure S2). Compared with Hom, Hem is characterized by earlier melting with a complex profile due to the formation of heteroduplexes [31]. The methylation status of the two genes in all samples is shown in Additional file 4 (Figure S3).

### Statistical analysis

The chi-square test was applied to examine the between-group differences for categorical variables, and Student’s *t* test was applied for continuous variables. Missing data less than 30% were addressed by multiple imputation. Logistic regression analysis was utilized to explore the correlation of DNA methylation and environmental exposures with GC. The odds ratios (ORs) and 95% confidence intervals (CIs) were adjusted by propensity scores (PSs). Gene-environment interactions were evaluated through multivariable logistic regression models. The combined effects of DNA methylation and environmental exposure were assessed by crossover analysis. An internal validation was further performed by repeating subsampling of two-thirds of the population 1000 times without replacement, and the average results from the internal validation datasets were calculated. Survival curves were generated using the Kaplan-Meier method. Cox regression models were applied to compute hazard ratios (HRs) and 95% CIs. The analyses were completed by SPSS 23.0. R-3.1.3 for Windows with PS matching 3.04 packages was applied for PS analysis. *P* < 0.05 was considered statistically significant.

## Results

### Characteristics of the subjects

Table 1 shows the demographic features of the study population. The differences in body mass index (BMI), occupation and monthly income between cases and controls were statistically significant (*P* < 0.05). In contrast to the controls, the GC patients exhibited a larger proportion of GC family history (*P* < 0.05).

**Table 1:** The basic demographic characteristics of the subjects

Variable		Cases (%)	Controls (%)	*P*
		*n* = 398	*n* = 397	
Sex	Male	301 (75.6)	300 (75.6)	0.984
	Female	97 (24.4)	97 (24.4)	
Age (mean ± SD)		58.22 ± 11.35	58.69 ± 10.50	0.549
	≥60	187 (47.0)	193 (48.6)	0.646
	< 60	211 (53.0)	204 (51.4)	
BMI (kg/m^2^)	≥24.00	125 (31.4)	194 (48.9)	< 0.001*
	< 24.00	273 (68.6)	203 (51.1)	
Occupation	White Collar	148 (37.2)	41 (10.3)	< 0.001*
	Blue Collar	250 (62.8)	356 (89.7)	
Monthly income (RMB/Per capita)	≥1000	258 (64.8)	213 (53.7)	0.001*
	< 1000	140 (35.2)	184 (46.3)	
Family history of GC	Yes	56 (14.1)	10 (2.5)	< 0.001*
	No	342 (85.9)	387 (97.5)	

*BMI* body mass index, *GC* gastric cancer

* Statistically significant

### The effects of environmental exposures on GC risk

The correlations of environmental exposures with GC risk are presented in Additional file 5 (Table S2). After backward conditional selection analysis, our data showed that infection with *H. pylori*, irregular diet, alcohol consumption, intake of freshwater fish, dairy products, food left overnight, salted food, fried food and drinking of unsanitary water (water from rivers and wells) could significantly increase the risk of GC (*P* < 0.05). Conversely, high intakes of green vegetables, garlic, eggs, refrigerated food and beef and mutton were correlated with a decreased risk of GC (*P* < 0.05). Consistent results were observed in the internal validation population (Additional file 6: Table S3).

### The impact of *ZNF331* and *WIF1* methylation on GC risk

Compared with Lm, *ZNF331* Hm was significantly associated with a decreased risk of GC after PS adjustment (OR = 0.580, 95% CI: 0.375–0.898, *P* = 0.015). However, *WIF1* methylation was not associated with GC risk (Table 2).

**Table 2:** Association between the methylation status of *ZNF331* and *WIF1* and GC risk

Methylation status		Case (%)	Control (%)	Crude OR(95% CI)	*P*	OR^a^ (95% CI)	*P*	OR^b^ (95% CI)	*P*
*ZNF331*	Hm	162 (43.8)	221 (60.9)	0.500 (0.373–0.672)	< 0.001*	0.585 (0.382–0.896)	0.014*	0.580 (0.375–0.898)	0.015*
	Lm	208 (56.2)	142 (39.1)	1.000		1.000		1.000	
*WIF1*	Hm	88 (24.0)	134 (35.7)	0.567 (0.412–0.781)	0.001*	0.715 (0.450–1.137)	0.157	0.714 (0.445–1.146)	0.162
	Lm	279 (76.0)	241 (64.3)	1.000		1.000		1.000	

*Lm* low methylation, *Hm* high methylation, *CI* confidence interval, *OR* odds ratio, *GC* gastric cancer

* Statistically significant

^a^ Adjusted for age, sex, BMI, monthly income, occupation, family history of GC, *H. pylori* infection, green vegetables, garlic, beef and mutton, freshwater fish, fried food, refrigerated food, egg, food left overnight, alcohol consumption, water, salted food, dairy products and irregular diet

^b^ Adjusted for propensity score of all variables

### Subgroup analysis

When the analysis was stratified by age, *ZNF331* Hm was marginally correlated with decreased GC risk among both the younger and older groups (OR = 0.523, 95% CI: 0.265–1.030, *P* = 0.061 and OR = 0.549, 95% CI: 0.296–1.018, *P* = 0.057, respectively). Subgroup analysis by sex showed that *ZNF331* Hm conferred a decreased GC risk only in males (OR = 0.577, 95% CI: 0.343–0.970, *P* = 0.038). In addition, subgroup analysis by *H. pylori* infection revealed a marginal association between *ZNF331* Hm and GC in the negative *H. pylori* infection group (OR = 0.474, 95% CI: 0.220–1.023, *P* = 0.057). For *WIF1*, there was a marginal correlation only in younger individuals (< 60 years, OR = 0.470, 95% CI: 0.219–1.007, *P* = 0.052) (Additional file 7: Table S4).

### The combined effects of *ZNF331* and *WIF1* (*ZW*) methylation on GC risk

To evaluate the combined effect between *ZNF331* and *WIF1* methylation, both *ZNF331* and *WIF1* Lm (*ZW* 1) was defined as a reference; likewise, either *ZNF331* or *WIF1* Hm was defined as *ZW* 2, and both *ZNF331* and *WIF1* Hm were regarded as *ZW* 3. The correlation between the newly defined *ZW* methylation and GC was explored. As presented in Table 3, *ZW* methylation was significantly correlated with the risk of GC after PS adjustment (OR = 0.419, 95% CI: 0.286–0.614, *P* < 0.001 and OR = 0.274, 95% CI: 0.158–0.474, *P* < 0.001 for *ZW* 2 and *ZW* 3, respectively). Moreover, the strength of the correlation significantly decreased (*P*-trend< 0.001).

**Table 3:** Combined effects of *ZNF331* and *WIF1* (*ZW*) methylation and GC risk

Methylation status		Case (%)	Control (%)	Crude OR(95% CI)	*P*	OR^a^ (95% CI)	*P*	OR^b^ (95% CI)	*P*	*P*-trend^c^
*ZW*	1	151 (44.5)	79 (23.2)	1.000		1.000		1.000		
	2	154 (45.4)	193 (56.6)	0.417 (0.296–0.589)	< 0.001*	0.471 (0.285–0.776)	0.003*	0.419 (0.286–0.614)	< 0.001*	
	3	34 (10.0)	69 (20.2)	0.258 (0.158–0.422)	< 0.001*	0.407 (0.202–0.821)	0.012*	0.274 (0.158–0.474)	< 0.001*	< 0.001*

1: Both *ZNF331* and *WIF1* were low methylated

2: One low and the other high

3: Both *ZNF331* and *WIF1* were high methylated

*CI* confidence interval, *OR* odds ratio, *GC* gastric cancer

* Statistically significant

^a^ Adjusted for age, sex, BMI, monthly income, occupation, family history of GC, *H. pylori* infection, green vegetables, garlic, beef and mutton, freshwater fish, fried food, refrigerated food, egg, food left overnight, alcohol consumption, water, salted food, dairy products and irregular diet

^b^ Adjusted for propensity score of all variables

^c^ The Cochran-Armitage trend test

### Correlations between environmental exposures and gene methylation

*H. pylori* infection decreased the risk of *ZNF331* methylation (OR = 0.645, 95% CI: 0.477–0.872, *P* = 0.004). Additionally, alcohol consumption and irregular diet were correlated with a decreased risk of *WIF1* methylation (OR = 0.724, 95% CI: 0.527–0.994, *P* = 0.045 and OR = 0.648, 95% CI: 0.443–0.946, *P* = 0.025, respectively) (Additional file 8: Table S5).

### The effects of gene-environment and gene-gene interactions on GC risk

*ZNF331* methylation and high intakes of green vegetables and garlic had significant combined effects on GC risk (OR = 0.073, 95% CI: 0.027–0.196, *P* < 0.001 and OR = 0.138, 95% CI: 0.080–0.238, *P* < 0.001, respectively) (Table 4), and the internal validation further demonstrated these combined effects (Additional file 9: Table S6). However, there were no interactions between *ZNF331* methylation and environmental exposure (Table 4 and Additional file 10: Table S7). *WIF1* methylation and intake of refrigerated food exhibited a significant combined effect (OR = 0.227, 95% CI: 0.136–0.380, *P* < 0.001), and their interaction displayed a synergistic effect on the risk of GC (OR = 2.588, 95% CI: 1.097–6.105, *P* = 0.030) (Additional file 11: Table S8). In addition, no interaction was observed between *ZNF331* and *WIF1* methylation and GC risk (*P* > 0.05) (Additional file 12: Table S9).

**Table 4:** Effects of the combination and interaction between environmental factors and *ZNF331* methylation status on GC risk

Environmental factors	*ZNF331* methylation status					
	Hm		Lm		Interactions	
	OR^a^ (95% CI)	*P*	OR^a^ (95% CI)	*P*	OR^b^ (95% CI)	*P*
Green vegetables (g/week)						
≥250	0.073 (0.027–0.196)	< 0.001*	0.126 (0.046–0.340)	< 0.001*	3.080 (0.911–10.406)	0.070
< 250	0.188 (0.058–0.603)	0.005*	1.000			
Garlic (times/week)						
≥1	0.138 (0.080–0.238)	< 0.001*	0.352 (0.211–0.586)	< 0.001*	0.680 (0.328–1.411)	0.300
< 1	0.576 (0.383–0.868)	0.008*	1.000			

*Lm* low methylation, *Hm* high methylation, *CI* confidence interval, *OR* odds ratio, *GC* gastric cancer

* Statistically significant

^a^ Combined effects adjusted for propensity score of age, sex, BMI, occupation, monthly income and family history of GC

^b^ Interactions adjusted for propensity score of age, sex, BMI, occupation, monthly income and family history of GC

### Gene methylation and GC prognosis

The associations of demographic and clinicopathological features with GC prognosis are shown in Additional files 13 and 14 (Tables S10 and S11, respectively). Tumour size, tumour node metastasis (TNM) stage, carcinoembryonic antigen (CEA) and carbohydrate antigen 19–9 (CA 19–9) levels were significantly correlated with GC prognosis after adjusting for age, sex and BMI (*P* < 0.05). Further backward conditional selection suggested that only TNM stage (HR = 2.518, 95% CI: 1.197–5.297, *P* = 0.015 and HR = 5.629, 95% CI: 2.855–11.102, *P* < 0.001 for TNM stages III and IV, respectively) and tumour size (HR = 1.518, 95% CI: 1.137–2.027, *P* = 0.005) were independent factors for GC prognosis (Additional file 15: Table S12).

The potential impact of *ZNF331* and *WIF1* methylation on the prognosis of GC was further investigated. However, no significant associations of methylation status of the two genes with GC prognosis were found (*P* > 0.05) (Table 5). The survival curves are shown in Additional file 16 (Figure S4). When the analysis was stratified by sex, a marginal correlation between *WIF1* methylation and GC prognosis was found after multivariable adjustment (HR = 0.643, 95% CI: 0.411–1.008, *P* = 0.054 and HR = 1.878, 95% CI: 0.952–3.705, *P* = 0.069 for males and females, respectively), whereas no association was found after PS adjustment. Stratified analyses according to age, *H. pylori* infection, TNM stage and tumour size suggested no significant association in any subgroup (*P* > 0.05) (Additional file 17: Table S13).

**Table 5:** Association between the methylation status of *ZNF331* and *WIF1* and GC prognosis

Methylation status		Case(%)	Crude HR(95%CI)	*P*	HR^a^ (95%CI)	*P*	HR^b^ (95%CI)	*P*
*ZNF331*	Hm	157 (45.1)	0.960 (0.713–1.293)	0.788	1.088 (0.799–1.483)	0.592	1.052 (0.759–1.457)	0.762
	Lm	191 (54.9)	1.000		1.000		1.000	
*WIF1*	Hm	84 (23.9)	0.892 (0.625–1.272)	0.528	0.852 (0.592–1.225)	0.386	0.830 (0.568–1.215)	0.338
	Lm	267 (76.1)	1.000		1.000		1.000	

*Lm* low methylation, *Hm* high methylation, *CI* confidence interval, *HR* hazard ratio, *GC* gastric cancer

^a^ Adjusted for age, sex, BMI, tumor size, TNM stage

^b^ Adjusted for propensity score of all variables

## Discussion

Aberrant DNA methylation, which induces abnormal expression of cancer-related genes, is one of the most common epigenetic mechanisms in tumour development and progression. It is known that DNA methylation changes during carcinogenesis are not confined to the target cells of the tumour tissue itself and that the immune system, such as leukocytes, may undergo specific methylation variations in the genome due to immune responses in early stages [32]. Since the sampling of peripheral blood is non-invasive and easy, assessing the DNA methylation status in PBLs may serve as a novel tool to determine cancer risks and prognoses. Therefore, we focused on the methylation of two tumour suppressor genes derived from PBLs and the impact on the risk and prognosis of GC in this population-based study.

Our study first demonstrated that subjects with *ZNF331* Hm had a significantly lower risk of GC, and the effect was confirmed in a PS adjusted model that included all the other variables in the study. This finding was intriguing in that the lower *ZNF331* promoter methylation level in GC cases is inconsistent with previous reports that *ZNF331* serves as a tumour suppressor gene that is hypermethylated and downregulated in several types of cancer tissues, including GC [14], colorectal cancer [15–17], and oesophageal squamous cell carcinoma [33]. However, it has been reported that leukocyte-derived DNA methylation changes at specific loci may not reflect those in target tissues [24], which means there was little correlation between the two origins. Some studies further hypothesized that DNA methylation changes in PBLs may arise from alterations in the leukocyte subpopulation, which was a response of immune surveillance to the appearance of tumours [34]. In addition to the possibility that DNA methylation exhibits high tissue specificity, it is also worth noting that the differentially methylated region in this study was located at − 701 to − 573 relative to the transcription start site (NM_001079907, hg19), which was different from the target regions in previous reports. Numerous studies have indicated that global DNA hypomethylation in PBLs, measured as the methylation status of repetitive sequences (such as LINE-1), is associated with GC risk [35, 36]. In addition, it was reported that hypomethylation of normally methylated promoters is associated with global hypomethylation and independent of frequent promoter hypermethylation [37]. We therefore hypothesized that significant global hypomethylation might disrupt certain protective mechanisms of methylated promoter regions and subsequently induce promoter hypomethylation. In addition, no differences in *WIF1* promoter methylation levels were found between GC cases and controls in this study, while previous reports have shown *WIF1* hypermethylation in circulating DNA of colorectal cancer patients [38, 39] and in tissue-derived DNA of several types of tumours, including GC [20]. Interestingly, in our previous work using the same detection method, we demonstrated expected hypermethylation of *WIF1* in colorectal tumour tissues [40], which to some extent indicated the tissue specificity of DNA methylation. In short, the methylation of *ZNF331* and *WIF1* we observed was limited to PBLs, which may not be extended to gastric tumour tissues. More efforts are needed to elucidate the underlying molecular mechanisms involved in these findings, especially for the nature and origin of DNA methylation in PBLs.

Epidemiological studies have suggested that environmental factors such as *H. pylori* infection, heavy alcohol consumption, cigarette smoking, salty food intake, and consumption of poorly preserved, pickled or contaminated foods are associated with GC [2, 41–43]. Environmental factors, including dietary habits, play important roles in carcinogenesis through the modification of DNA methylation [11]. Thus, the interactions between gene methylation and dietary factors on the risk of GC were explored in the present work, and the combined effects were also taken into account. Confounders were controlled by PS adjustment. Substantial evidence has strongly suggested that GC risk may be decreased with a high intake of vegetables [44]. As expected, significant combined effects between *ZNF331* methylation and the intake of green vegetables were observed in this study. Green vegetables are rich in polyphenol compounds, which possess anti-cancer effects through alterations in DNA methylation, histone modifications and miRNA expression [45]. Additionally, increased consumption of garlic has been reported to reduce the risk of GC [46], which may explain the combined effects of garlic consumption and *ZNF331* methylation on GC risk. It has been found that organosulfur compounds that naturally exist in garlic could inhibit benzo [a]pyrene-induced neoplasia in the forestomach of mice [47]. Another study has shown that S-allylcysteine derived from garlic could inhibit the expression of DNA methyltransferase 1 and then induce global DNA hypomethylation in human ovarian cancer cells [48]. However, interactions between the intake of green vegetables and garlic and *ZNF331* methylation were not found in this study.

Growing evidence has revealed that there is age-related accumulation of DNA methylation during tumourigenesis [49] and that DNA methylation patterns in blood between men and women exhibit considerable differences [50]. In addition, *H. pylori* and the inflammatory response it triggers may facilitate carcinogenesis in gastric epithelial cells by inducing dysregulation of DNA methylation in the promoter regions of various genes [51, 52]. Given the points mentioned above, we performed subgroup analyses according to age, sex and *H. pylori* infection. The results indicated that *ZNF331* methylation was significantly correlated with GC only in males, which to some extent illustrates the sex-related methylation differences. There were also marginal correlations between *ZNF331* methylation and GC within both the younger and older groups, *WIF1* methylation and GC risk in the younger group, and *ZNF331* methylation and GC risk in the *H. pylori*-negative group.

The prognostic effect of *ZNF331* and *WIF1* methylation was also evaluated in the present work. The results concluded that tumour size and tumour stage could independently influence GC prognosis. It has been reported that colorectal cancer patients with *ZNF331* methylation have poor overall survival [16, 17], and *WIF1* methylation is negatively correlated with prognosis in oesophageal cancer [22]. However, no association was found between candidate gene methylation and GC prognosis.

MS-HRM has been proposed to be a rapid and cost-effective way to measure methylation levels in a large panel of samples, and the detection limit can be as low as 0.1% of gene methylation [53–56]. Moreover, our previous studies have demonstrated the stability and reliability of the MS-HRM assay, which is feasible for methylation detection in peripheral blood, although the methylation levels measured by MS-HRM were relatively lower than those measured by pyrosequencing [57, 58]. It is also worth noting that the potential nondifferential misclassification bias due to the underestimation of gene methylation levels may have resulted in smaller but still significant effect sizes [59–61]. Nevertheless, we recognized the limitations of the MS-HRM method used in this study that it can only give a semiquantitative estimation of the DNA methylation level, and the results estimated from heterogeneously methylated samples are largely qualitative. Thus, further validations by sequencing methodologies or other available techniques are necessary for future work.

This study also has several other limitations. First, we could not elucidate the chronological order of gene methylation and GC due to the retrospective nature of this study. Second, recall bias may have affected our results, although efforts were made to minimize this bias. Third, dietary factors were collected by frequency rather than quantity, which might influence the efficiency of the analysis.

## Conclusion

In summary, this study uncovered that *ZNF331* methylation in PBLs was correlated with the risk of GC. The combined effects between *ZNF331* methylation and high intakes of green vegetables and garlic might decrease the risk of GC.

## Supplementary Information

**Additional file 1: Table S1.** Detailed information of the amplified regions and primer sequences and the reaction conditions for methylation-sensitive high-resolution melting (MS-HRM) assay.

**Additional file 2: Figure S1.** Receiver operating characteristic curve and the corresponding area under the curve (AUC) analyses of *ZNF331* methylation on gastric cancer risk.

**Additional file 3: Figure S2.** The normalized melting curves and melting peaks of homogeneous methylation (Hom) and heterogeneous methylation (Hem) for *WIF1*.

**Additional file 4: Figure S3.** The distribution of *ZNF331* (a) and *WIF1* (b) methylation status in GC cases and controls.

**Additional file 5: Table S2.** Association between environmental factors and GC risk.

**Additional file 6: Table S3.** Multivariate analysis and internal validation of the association between environmental factors and GC risk.

**Additional file 7: Table S4.** Association between the methylation status of genes and GC risk by stratified analysis.

**Additional file 8: Table S5.** Association between *ZNF331* and *WIF1* methylation and environmental factors.

**Additional file 9: Table S6.** Effects of the combination between environmental factors and *ZNF331* methylation status on GC risk in the whole population and internal validation datasets.

**Additional file 10: Table S7.** Effects of the combination and interaction between environmental factors and *ZNF331* methylation status on GC risk.

**Additional file 11: Table S8.** Effects of the combination and interaction between environmental factors and *WIF1* methylation status on GC risk.

**Additional file 12: Table S9.** Effects of the combination and interaction between *ZNF331* and *WIF1* methylation on GC risk.

**Additional file 13: Table S10.** Association between demographic characteristics and GC prognosis.

**Additional file 14: Table S11.** Association between clinical characteristics and GC prognosis.

**Additional file 15: Table S12.** Multivariate analysis of GC prognosis.

**Additional file 16: Figure S4.** Survival curves of the association between *ZNF331* (a) and *WIF1* (b) methylation and GC prognosis.

**Additional file 17: Table S13.** Association between the methylation status of genes and GC prognosis by stratified analysis.

## Data Availability

The datasets generated and/or analysed during the current study are not publicly available because the project is currently under study but are available from the corresponding author upon reasonable request.

## Abbreviations

PBL: Peripheral blood leukocyte
GC: Gastric cancer
*ZNF331*: Zinc-finger protein 331
*WIF1*: Wnt-inhibitory factor-1
MS-HRM: Methylation-sensitive high-resolution melting
PS: Propensity score
Hm: High methylation
Lm: Low methylation
Hom: Homogeneous methylation
Hem: Heterogeneous methylation
ORs: Odds ratios
CIs: Confidence intervals
HRs: Hazard ratios
BMI: Body mass index
TNM: Tumour node metastasis
CEA: Carcinoembryonic antigen
CA 19–9: Carbohydrate antigen 19–9
