# Supplementary material for: Association of ZNF331 and WIF1 methylation in peripheral blood leukocytes with the risk and prognosis of gastric cancer
Source: BMC Cancer. 2021 May 15;21:551. doi: 10.1186/s12885-021-08199-4 (PMC8126111; doi:10.1186/s12885-021-08199-4)
Supplement: Supplementary file 1 — Additional file 1: Table S1. Detailed information of the amplified regions and primer sequences and the reaction conditions for methylation-sensitive high-resolution melting (MS-HRM) assay. [file 12885_2021_8199_MOESM1_ESM.docx]

**Association of *ZNF331* and *WIF1* methylation in peripheral blood leukocytes with the risk and prognosis of gastric cancer**

Chuang Nie^1^, Xu Han^1^, Rongrong Wei^1^, Anastasiia Leonteva^1^, Jia Hong^1^, Xinyu Du^1^, Jing Wang^1^, Lin Zhu^1^, Yashuang Zhao^1^, Yingwei Xue^2^, Haibo Zhou^1^*, Wenjing Tian^1^*

^1^ Department of Epidemiology, College of Public Health, Harbin Medical University, Harbin, 197 Xuefu Road, Harbin 150081, Heilongjiang Province, P. R. China.

^2^ Department of Gastroenterological Surgery, Third Affiliated Hospital of Harbin Medical University, 150 Haping Road, Harbin 150081, Heilongjiang Province, P. R. China.

***Corresponding authors:**

Wenjing Tian, M.D., Ph.D., Department of Epidemiology, College of Public Health, Harbin Medical University, Postal address: 197 Xuefu Road, Harbin 150081, Heilongjiang Province, P. R. China. Tel: +86-451-87502685; Fax: +86-451-87502885; E-mail: twj8267@sina.com; ORCID: https://orcid.org/0000-0002-0449-6643.

Haibo Zhou, M.D., Ph.D., Department of Epidemiology, College of Public Health, Harbin Medical University, Postal address: 197 Xuefu Road, Harbin 150081, Heilongjiang Province, P. R. China; Tel: +86-451-86611797; Fax: +86-451-87502885; E-mail: youdeng23@163.com; ORCID: https://orcid.org/0000-0001-5991-0840.

**Table S1** Detailed information of the amplified regions and primer sequences and the reaction conditions for methylation-sensitive high-resolution melting (MS-HRM) assay

| Gene | Locus (GRCh37/hg19 Feb 2009) | | Strand | Length (bp) | CpG number | Primer sequence for MS-HRM assay (5' to 3') | Cycling time (sec) | Annealing temperature (℃) | Melting temperature (℃) | ℃/step | Cycles |
| --- | --- | --- | --- | --- | --- | --- | --- | --- | --- | --- | --- |
| *ZNF331* | chr19: 54057860-54057989 | | + | 128bp | 14 | F:5' CGTAAGCGTTATTGGGGGTGAT 3' | 10-30-20 | 57 | 70-94 | 0 | 50 |
|  |  |  |  |  |  | R:5' CCGTACCCGTCTCCCCTTA 3' |  |  |  |  |  |
| *WIF1* | chr12: 65515413-65515533 | | - | 121bp | 10 | F:5' GAGGTGGCGAGTGATGTTTTAGG 3' | 10-30-20 | 66-60 | 65-90 | 0.1 | 60 |
|  |  |  |  |  |  | R:5' AACCCCCGAAACTACATTCACAATA 3' |  |  |  |  |  |
| Gene | DNA methylated standards | Sequence (5' to 3') | | | | | | | | | |
| *ZNF331* | 100% methylated DNA | cgtaagcgttattgggggtgatgggggtcgtgttcggtgcgtttttgtatcggtgacgtaatcgttgtgttttcgttagtcgcgtaggtgagattggcgtttatggttttaaggggagacgggtacgg | | | | | | | | | |
|  | 0% methylated DNA | cgcaagcgtcattgggggtgatgggggccgtgctcggtgcgcttctgcaccggtgacgcaaccgctgtgtctccgccagccgcgcaggtgagattggcgcccatggctccaaggggagacgggcacgg | | | | | | | | | |
| *WIF1* | 100% methylated DNA | gaggtggcgagtgatgttttaggggtttttgagtgttttttttcgggttcgttagttttatacgtttatttcgcgggcgttttattgggcgtatcgtattgtgaatgtagtttcgggggtt | | | | | | | | | |
|  | 0% methylated DNA | gaggtggcgagtgatgtcccaggggtctctgagtgcccttctccgggtccgccagccctacacgcccacttcgcgggcgctccactgggcgcaccgcactgtgaatgcagcctcgggggtc | | | | | | | | | |

Note: The reaction volume was 5 µL, including 2.5µl of LightCycler 480 High Resolution Melting Master Mix (Roche), 0.5µl of sodium bisulfite-modified template DNA, 0.1µl of each forward and reverse primer, 0.6µl of MgCl2, and 1.2µl of PCR-grade water.
