# Supplementary material for: Association of ZNF331 and WIF1 methylation in peripheral blood leukocytes with the risk and prognosis of gastric cancer
Source: BMC Cancer. 2021 May 15;21:551. doi: 10.1186/s12885-021-08199-4 (PMC8126111; doi:10.1186/s12885-021-08199-4)
Supplement: Supplementary file 2 — Additional file 2: Figure S1. Receiver operating characteristic curve and the corresponding area under the curve (AUC) analyses of ZNF331 methylation on gastric cancer risk. [file 12885_2021_8199_MOESM2_ESM.docx]

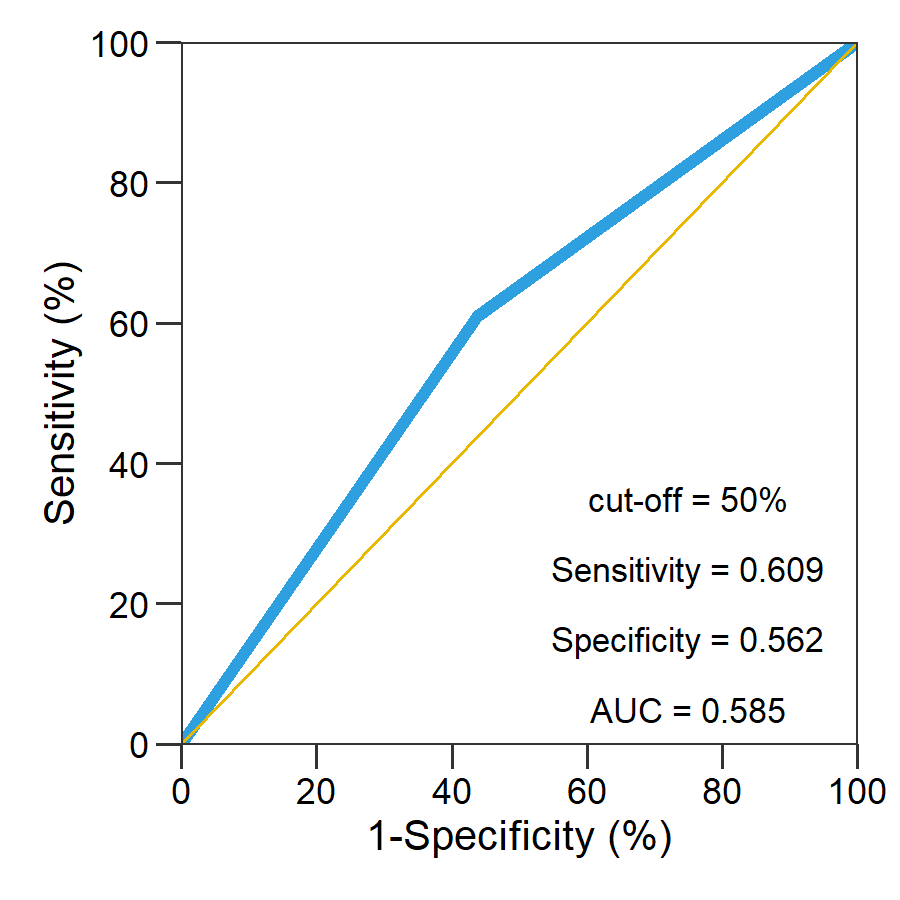


**Figure S1.** Receiver operating characteristic curve and the corresponding area under the curve (AUC) analyses of *ZNF331* methylation on gastric cancer risk.
