# Supplementary material for: Association of ZNF331 and WIF1 methylation in peripheral blood leukocytes with the risk and prognosis of gastric cancer
Source: BMC Cancer. 2021 May 15;21:551. doi: 10.1186/s12885-021-08199-4 (PMC8126111; doi:10.1186/s12885-021-08199-4)
Supplement: Supplementary file 3 — Additional file 3: Figure S2. The normalized melting curves and melting peaks of homogeneous methylation (Hom) and heterogeneous methylation (Hem) for WIF1. [file 12885_2021_8199_MOESM3_ESM.docx]

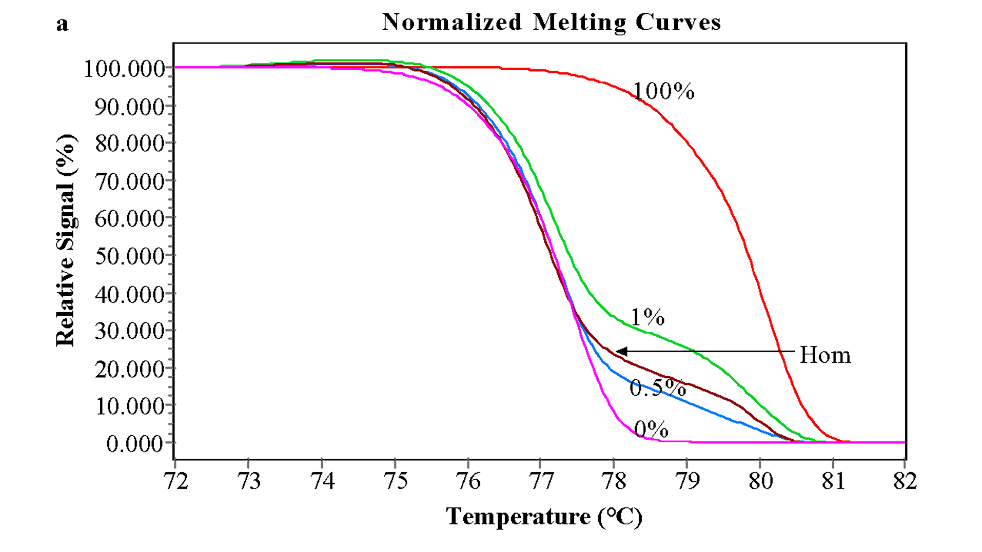

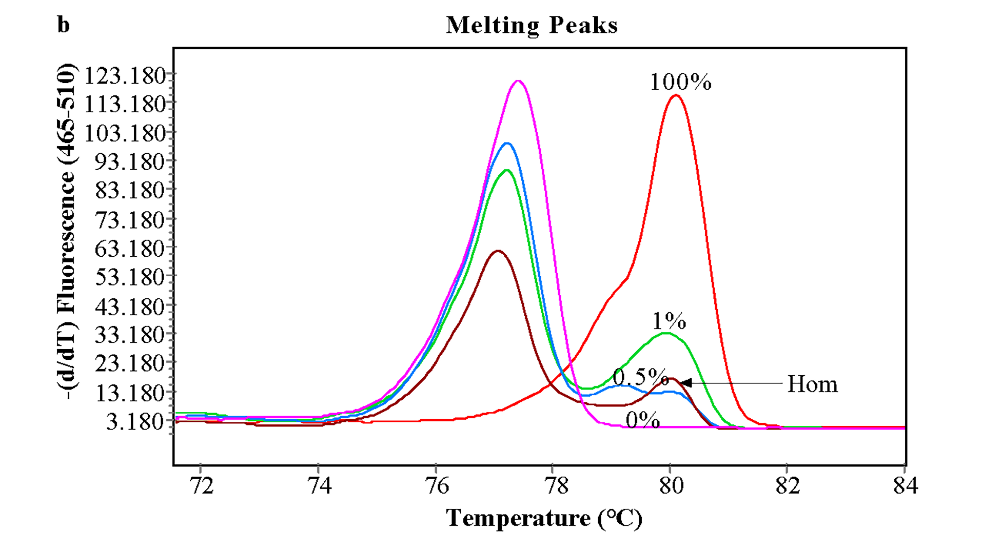


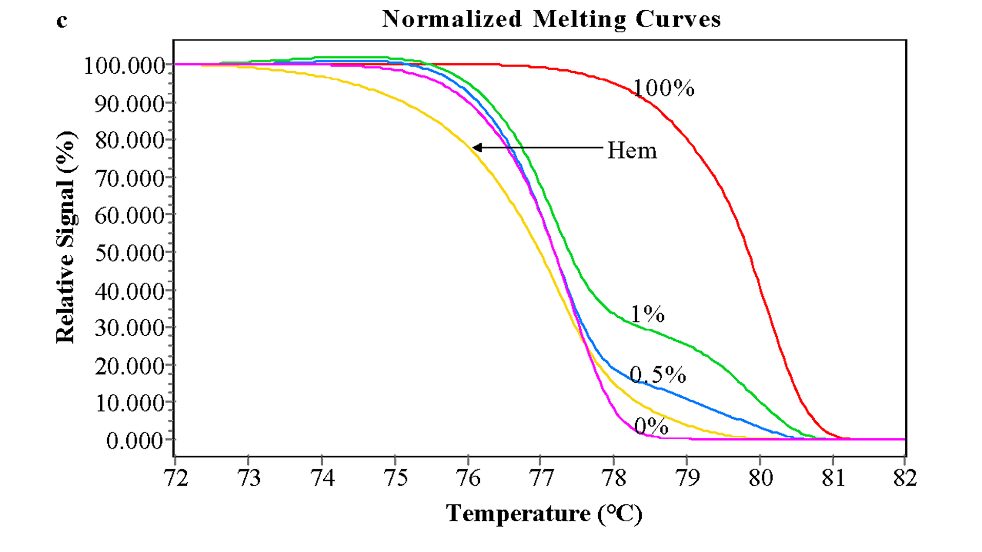

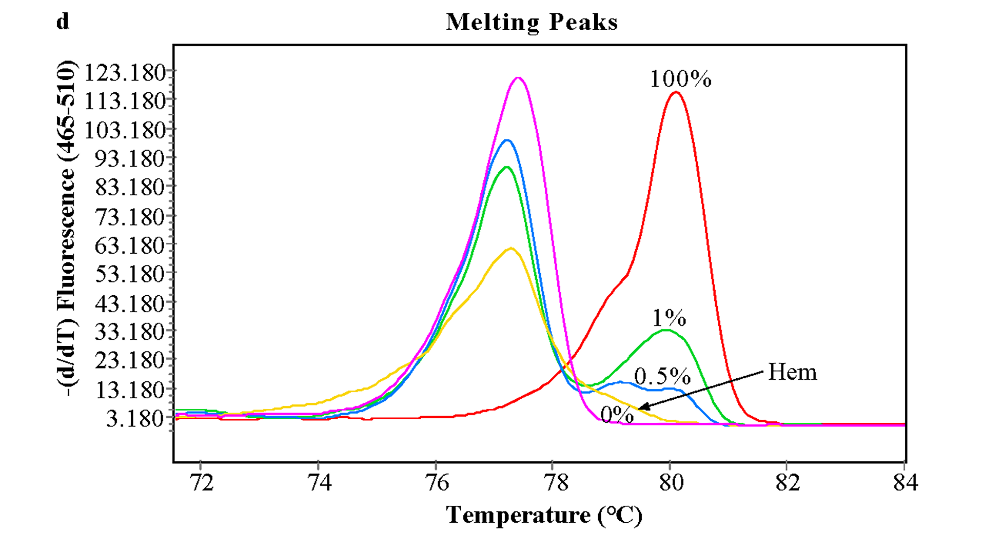


**Figure S2.** The normalized melting curves and melting peaks of homogeneous methylation (Hom) and heterogeneous methylation (Hem) for *WIF1*. **(a)** Normalized melting curves of methylated DNA standards and homogeneous methylation (Hom) for *WIF1*. **(b)** Melting peaks of methylated DNA standards and Hom were generated by taking the negative derivative (d) of the melting curve data divided by the derivative with respect to time‐(d/dT). **(c)** Normalized melting curves of methylated DNA standards and heterogeneous methylation (Hem) for *WIF1*. **(d)** Melting peaks of methylated DNA standards and Hem were generated by taking the negative derivative (d) of the melting curve data divided by the derivative with respect to time‐(d/dT).
