# Supplementary figures and images for: Association of ZNF331 and WIF1 methylation in peripheral blood leukocytes with the risk and prognosis of gastric cancer
Source: BMC Cancer. 2021 May 15;21:551. doi: 10.1186/s12885-021-08199-4 (PMC8126111; doi:10.1186/s12885-021-08199-4)

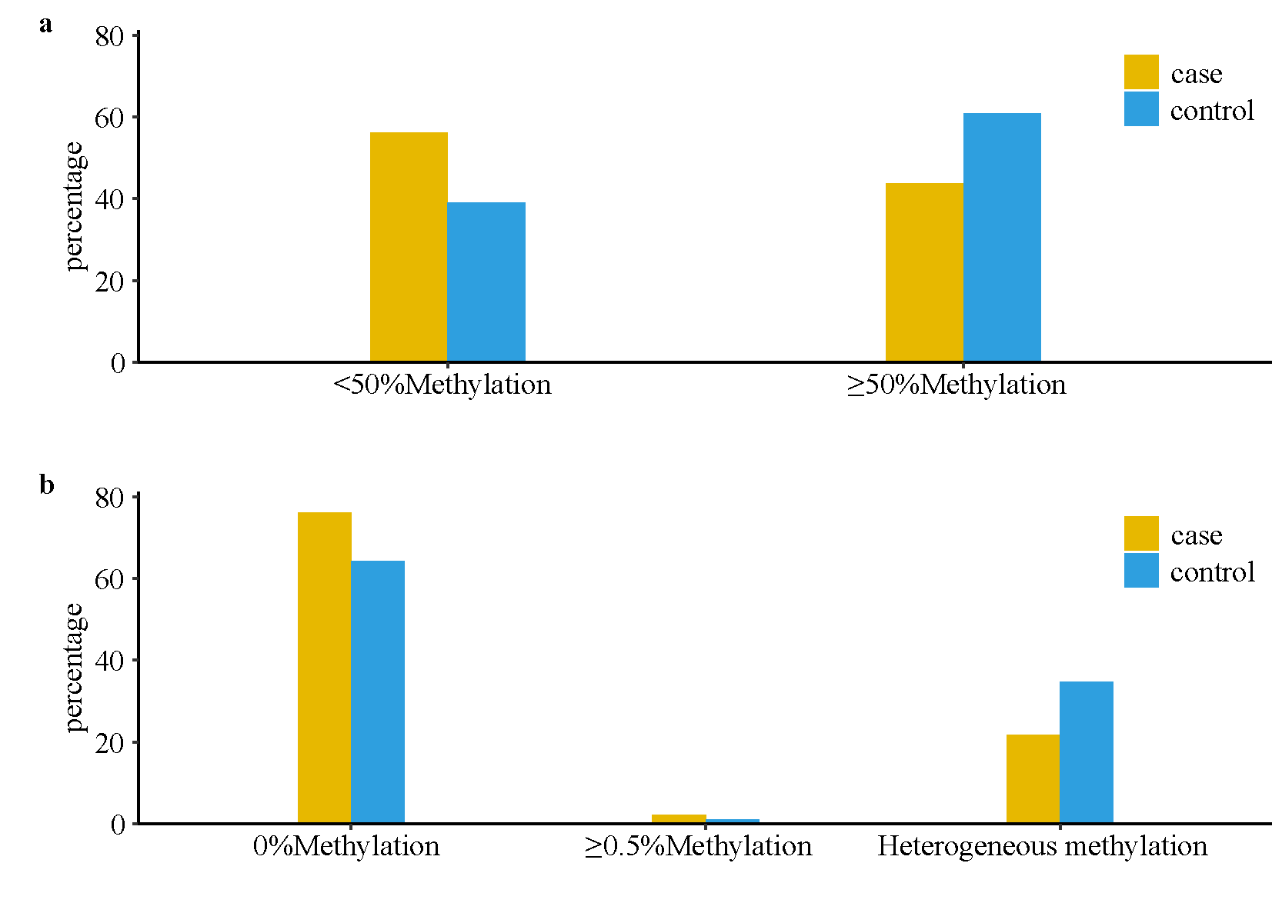


2.2%

1.1%

34.7%

21.8%

76.0%

64.2%

56.2%

43.8%

60.9%

39.1%

**Figure S3.** The distribution of *ZNF331* **(a)** and *WIF1* **(b)** methylation status in GC cases and controls.

Supplement: Supplementary file 4 — Additional file 4: Figure S3. The distribution of ZNF331 (a) and WIF1 (b) methylation status in GC cases and controls. [file 12885_2021_8199_MOESM4_ESM.docx]
