# Supplementary material for: Association of ZNF331 and WIF1 methylation in peripheral blood leukocytes with the risk and prognosis of gastric cancer
Source: BMC Cancer. 2021 May 15;21:551. doi: 10.1186/s12885-021-08199-4 (PMC8126111; doi:10.1186/s12885-021-08199-4)
Supplement: Supplementary file 5 — Additional file 5: Table S2. Association between environmental factors and GC risk. [file 12885_2021_8199_MOESM5_ESM.docx]

**Table S2** Association between environmental factors and GC risk

| Environmental factors |  | | Case (%) | | Control (%) | | Crude OR (95% CI) | | *P* | | OR^a^ (95% CI) | | *P* | |
| --- | --- | --- | --- | --- | --- | --- | --- | --- | --- | --- | --- | --- | --- | --- |
| Alcohol consumption | Yes | | 232(58.3) | | 192(48.4) | | 1.492(1.124-1.982) | | 0.006 | | 1.592(1.158-2.189) | | 0.004 | |
|  | No | | 166(41.7) | | 205(51.6) | | 1.000 | |  | | 1.000 | |  | |
| Bean products (times/week) | ≥3 | | 186(46.7) | | 229(57.7) | | 0.638(0.480-0.848) | | 0.002 | | 0.627(0.459-0.856) | | 0.003 | |
|  | <3 | | 212(53.3) | | 168(42.3) | | 1.000 | |  | | 1.000 | |  | |
| Beef and mutton (g/week) | ≥250 | | 41(10.3) | | 79(19.9) | | 0.462(0.306-0.697) | | <0.001 | | 0.508(0.324-0.798) | | 0.003 | |
|  | <250 | | 357(89.7) | | 318(80.1) | | 1.000 | |  | | 1.000 | |  | |
| Chicken (g/week) | ≥250 | | 55(13.8) | | 81(20.4) | | 0.624(0.423-0.921) | | 0.017 | | 0.783(0.512-1.196) | | 0.257 | |
|  | <250 | | 343(86.2) | | 316(79.6) | | 1.000 | |  | | 1.000 | |  | |
| Irregular diet | Yes | | 146(36.7) | | 62(15.6) | | 3.135(2.227-4.414) | | <0.001 | | 3.279(2.256-4.765) | | <0.001 | |
|  | No | | 252(63.3) | | 335(84.4) | | 1.000 | |  | | 1.000 | |  | |
| Eating speed | | Fast | | 195(49.0) | | 173(43.6) | | 1.242(0.938-1.646) | | 0.130 | | 1.348(0.986-1.844) | | 0.061 |
|  | | Slow | | 203(51.0) | | 224(56.4) | | 1.000 | |  | | 1.000 | |  |
| Egg (g/week) | | ≥350 | | 89(22.4) | | 126(31.7) | | 0.618(0.446-0.855) | | 0.004 | | 0.566(0.395-0.811) | | 0.002 |
|  | | <350 | | 309(77.6) | | 271(68.3) | | 1.000 | |  | | 1.000 | |  |
| Food left overnight | | ≥1 | | 318(79.9) | | 264(66.5) | | 2.021(1.455-2.808) | | <0.001 | | 2.255(1.559-3.262) | | <0.001 |
| (times/week) | | <1 | | 80(20.1) | | 133(33.5) | | 1.000 | |  | | 1.000 | |  |
| Freshwater fish (times/week) | | ≥1 | | 172(43.2) | | 58(14.6) | | 4.426(3.144-6.232) | | <0.001 | | 4.151(2.861-6.024) | | <0.001 |
|  | | <1 | | 226(56.8) | | 339(85.4) | | 1.000 | |  | | 1.000 | |  |
| Fried food (times/week) | | ≥1 | | 208(52.3) | | 173(43.6) | | 1.420(1.072-1.882) | | 0.015 | | 1.432(1.046-1.962) | | 0.025 |
|  | | <1 | | 190(47.7) | | 224(56.4) | | 1.000 | |  | | 1.000 | |  |
| Fruits (g/week) | | ≥1000 | | 155(38.9) | | 169(42.6) | | 0.868(0.652-1.156) | | 0.332 | | 0.811(0.591-1.113) | | 0.194 |
|  | | <1000 | | 243(61.1) | | 228(57.4) | | 1.000 | |  | | 1.000 | |  |
| Garlic (times/week) | | ≥1 | | 85(21.4) | | 191(48.1) | | 0.293(0.214-0.401) | | <0.001 | | 0.296(0.209-0.420) | | <0.001 |
|  | | <1 | | 313(78.6) | | 206(51.9) | | 1.000 | |  | | 1.000 | |  |
| Environmental factors | |  | | Case (%) | | Control (%) | | Crude OR (95% CI) | | *P* | | OR^a^ (95% CI) | | *P* |
| Green vegetables (g/week) | | ≥250 | | 317(79.6) | | 372(93.7) | | 0.259(0.160-0.418) | | <0.001 | | 0.257(0.153-0.432) | | <0.001 |
|  | | <250 | | 81(20.4) | | 25(6.3) | | 1.000 | |  | | 1.000 | |  |
| Hot food | | Yes | | 178(44.7) | | 186(46.9) | | 0.920(0.692-1.223) | | 0.564 | | 0.912(0.667-1.247) | | 0.564 |
|  | | No | | 220(55.3) | | 211(53.1) | | 1.000 | |  | | 1.000 | |  |
| Marine product (times/week) | | ≥1 | | 47(11.8) | | 47(11.8) | | 0.994(0.642-1.539) | | 0.980 | | 0.681(0.414-1.122) | | 0.131 |
|  | | <1 | | 351(88.2) | | 350(88.2) | | 1.000 | |  | | 1.000 | |  |
| Pork (g/week) | | ≥250 | | 250(62.8) | | 236(59.4) | | 1.152(0.857-1.549) | | 0.347 | | 1.158(0.839-1.598) | | 0.371 |
|  | | <250 | | 148(37.2) | | 161(40.6) | | 1.000 | |  | | 1.000 | |  |
| Refrigerated food | | Yes | | 252(63.3) | | 317(79.8) | | 0.436(0.316-0.602) | | <0.001 | | 0.389(0.273-0.554) | | <0.001 |
|  | | No | | 146(36.7) | | 80(20.2) | | 1.000 | |  | | 1.000 | |  |
| Salted food | | Yes | | 215(54.0) | | 141(35.5) | | 2.145(1.611-2.855) | | <0.001 | | 1.996(1.456-2.737) | | <0.001 |
|  | | No | | 183(46.0) | | 256(64.5) | | 1.000 | |  | | 1.000 | |  |
| Tea | | Yes | | 113(28.4) | | 132(33.2) | | 0.804(0.592-1.091) | | 0.161 | | 0.844(0.603-1.181) | | 0.322 |
|  | | No | | 285(71.6) | | 265(66.8) | | 1.000 | |  | | 1.000 | |  |
| Water | | River-water and well-water | | 112(28.1) | | 85(21.4) | | 1.442(1.040-2.000) | | 0.028 | | 1.816(1.265-2.608) | | 0.001 |
|  | | Tap water and mineral-water | | 286(71.9) | | 312(78.6) | | 1.000 | |  | | 1.000 | |  |
| Dairy products (times/week) | | ≥1 | | 254(63.8) | | 177(44.6) | | 2.195(1.646-2.927) | | <0.001 | | 1.787(1.304-2.449) | | <0.001 |
|  | | <1 | | 144(36.2) | | 220(55.4) | | 1.000 | |  | | 1.000 | |  |
| *H. pylori* infection | | Positive | | 249(62.6) | | 189(47.6) | | 1.830(1.367-2.450) | | <0.001 | | 1.964(1.406-2.742) | | <0.001 |
|  | | Negative | | 149(37.4) | | 208(52.4) | | 1.000 | |  | | 1.000 | |  |
| Smoking | | Yes | | 245(61.6) | | 207(52.1) | | 1.469(1.105-1.951) | | 0.008 | | 1.602(1.167-2.199) | | 0.004 |
|  | | No | | 153(38.4) | | 190(47.9) | | 1.000 | |  | | 1.000 | |  |

CI, confidence interval; OR odds ratio; GC, gastric cancer.

^a^ Adjusted for propensity score of age, sex, BMI, occupation, monthly income and family history of GC.
