# Supplementary material for: Association of ZNF331 and WIF1 methylation in peripheral blood leukocytes with the risk and prognosis of gastric cancer
Source: BMC Cancer. 2021 May 15;21:551. doi: 10.1186/s12885-021-08199-4 (PMC8126111; doi:10.1186/s12885-021-08199-4)
Supplement: Supplementary file 6 — Additional file 6: Table S3. Multivariate analysis and internal validation of the association between environmental factors and GC risk. [file 12885_2021_8199_MOESM6_ESM.docx]

**Table S3** Multivariate analysis and internal validation of the association between environmental factors and GC risk

| Environmental factors | Whole population (N=795) | | | |  | Internal validation population (N=530) |
| --- | --- | --- | --- | --- | --- | --- |
|  | *β-coefficient* | SE | *P* | OR(95% CI) |  | OR^a^ (95%CI)^b^ |
| *H. pylori* infection | 0.635 | 0.204 | 0.002 | 1.887(1.265-2.815) | | 2.091(1.523-2.882) |
| Irregular diet | 1.422 | 0.242 | <0.001 | 4.145(2.580-6.660) | | 4.546(3.194-6.640) |
| Salted food | 0.755 | 0.207 | <0.001 | 2.127(1.417-3.191) | | 2.069(1.568-2.886) |
| Green vegetables (g/week) | -1.464 | 0.331 | <0.001 | 0.231(0.121-0.443) | | 0.227(0.139-0.357) |
| Garlic (times/week) | -1.413 | 0.229 | <0.001 | 0.243(0.155-0.382) | | 0.232(0.154-0.330) |
| Beef and mutton (g/week) | -0.905 | 0.325 | 0.005 | 0.405(0.214-0.765) | | 0.401(0.251-0.641) |
| Freshwater fish (times/week) | 1.633 | 0.240 | <0.001 | 5.120(3.197-8.201) | | 5.401(3.858-8.263) |
| Dairy products (times/week) | 1.293 | 0.232 | <0.001 | 3.643(2.313-5.736) | | 3.856(2.771-5.830) |
| Fried food (times/week) | 0.548 | 0.209 | 0.009 | 1.730(1.149-2.605) | | 1.742(1.263-2.392) |
| Refrigerated food | -1.390 | 0.248 | <0.001 | 0.249(0.153-0.405) | | 0.234(0.153-0.330) |
| Alcohol consumption | 0.911 | 0.248 | <0.001 | 2.487(1.530-4.043) | | 2.106(1.482-3.015) |
| Water | 0.841 | 0.248 | 0.001 | 2.319(1.428-3.767) | | 2.296(1.628-3.428) |
| Egg (g/week) | -0.574 | 0.231 | 0.013 | 0.563(0.358-0.886) | | 0.589(0.407-0.814) |
| Food left overnight (times/week) | 1.187 | 0.254 | <0.001 | 3.278(1.992-5.392) | | 3.488(2.424-5.160) |
| Sex | 0.528 | 0.289 | 0.068 | 1.695(0.962-2.985) | | 1.419(0.931-2.277) |
| Age | -0.222 | 0.221 | 0.314 | 0.801(0.519-1.235) | | 0.772(0.541-1.086) |
| BMI (kg/m^2^) | -0.722 | 0.211 | 0.001 | 0.486(0.321-0.734) | | 0.502(0.366-0.673) |
| Monthly income (RMB/Per capita) | 0.476 | 0.221 | 0.031 | 1.609(1.044-2.480) | | 1.494(1.072-2.055) |
| Occupation | -1.558 | 0.260 | <0.001 | 0.210(0.126-0.350) | | 0.178(0.109-0.274) |
| Family history of GC | 1.875 | 0.426 | <0.001 | 6.522(2.831-15.025) | | 10.555(6.204-21.106) |

CI, confidence interval; OR, odds ratio; GC, gastric cancer; SE, standard error.

^a^ Median odds ratio was derived from the internal validation datasets generated by repeating 1000 times of subsampling without replacement.

^b^ Intervals from the 2.5th to the 97.5th percentile.
