# Supplementary material for: Association of ZNF331 and WIF1 methylation in peripheral blood leukocytes with the risk and prognosis of gastric cancer
Source: BMC Cancer. 2021 May 15;21:551. doi: 10.1186/s12885-021-08199-4 (PMC8126111; doi:10.1186/s12885-021-08199-4)
Supplement: Supplementary file 7 — Additional file 7: Table S4. Association between the methylation status of genes and GC risk by stratified analysis. [file 12885_2021_8199_MOESM7_ESM.docx]

**Table S4** Association between the methylation status of genes and GC risk by stratified analysis

| Gene | | <60 years | | |  | ≥60 years | | |
| --- | --- | --- | --- | --- | --- | --- | --- | --- |
|  |  | OR | 95% CI | *P* |  | OR | 95% CI | *P* |
| *ZNF331* | Hm | 0.523 | 0.265-1.030 | 0.061 |  | 0.549 | 0.296-1.018 | 0.057 |
|  | Lm | 1.000 |  |  |  | 1.000 |  |  |
| *WIF1* | Hm | 0.470 | 0.219-1.007 | 0.052 |  | 0.833 | 0.434-1.598 | 0.582 |
|  | Lm | 1.000 |  |  |  | 1.000 |  |  |
| Gene | | Male | | |  | Female | | |
|  |  | OR | 95% CI | *P* |  | OR | 95% CI | *P* |
| *ZNF331* | Hm | 0.577 | 0.343-0.970 | 0.038 |  | 0.534 | 0.194-1.468 | 0.224 |
|  | Lm | 1.000 |  |  |  | 1.000 |  |  |
| *WIF1* | Hm | 0.691 | 0.394-1.213 | 0.198 |  | 0.792 | 0.257-2.446 | 0.685 |
|  | Lm | 1.000 |  |  |  | 1.000 |  |  |
| Gene | | *H. pylori* negative | | |  | *H. pylori* positive | | |
|  |  | OR | 95% CI | *P* |  | OR | 95% CI | *P* |
| *ZNF331* | Hm | 0.474 | 0.220-1.023 | 0.057 |  | 0.780 | 0.435-1.400 | 0.405 |
|  | Lm | 1.000 |  |  |  | 1.000 |  |  |
| *WIF1* | Hm | 0.734 | 0.325-1.658 | 0.456 |  | 0.658 | 0.352-1.230 | 0.190 |
|  | Lm | 1.000 |  |  |  | 1.000 |  |  |

Lm, low methylation; Hm, high methylation; CI, confidence interval; OR, odds ratio; GC, gastric cancer. OR adjusted for propensity score of all variables except stratified factors.
