# Supplementary material for: Association of ZNF331 and WIF1 methylation in peripheral blood leukocytes with the risk and prognosis of gastric cancer
Source: BMC Cancer. 2021 May 15;21:551. doi: 10.1186/s12885-021-08199-4 (PMC8126111; doi:10.1186/s12885-021-08199-4)
Supplement: Supplementary file 8 — Additional file 8: Table S5. Association between ZNF331 and WIF1 methylation and environmental factors. [file 12885_2021_8199_MOESM8_ESM.docx]

**Table S5** Association between *ZNF331* and *WIF1* methylation and environmental factors

| Environmental factors |  | *ZNF331* methylation | |  | *WIF1* methylation | |
| --- | --- | --- | --- | --- | --- | --- |
|  |  | OR^a^ (95% CI) | *P* |  | OR^a^ (95% CI) | *P* |
| Alcohol consumption | Yes | 1.095(0.816-1.468) | 0.546 |  | 0.724(0.527-0.994) | 0.045 |
|  | No | 1.000 |  |  | 1.000 |  |
| Bean products (times/week) | ≥3 | 0.929(0.692-1.246) | 0.623 |  | 1.124(0.818-1.545) | 0.470 |
|  | <3 | 1.000 |  |  | 1.000 |  |
| Beef and mutton (g/week) | ≥250 | 1.044(0.694-1.570) | 0.837 |  | 0.842(0.533-1.332) | 0.463 |
|  | <250 | 1.000 |  |  | 1.000 |  |
| Chicken (g/week) | ≥250 | 1.295(0.864-1.942) | 0.211 |  | 0.679(0.434-1.061) | 0.089 |
|  | <250 | 1.000 |  |  | 1.000 |  |
| Irregular diet | Yes | 0.781(0.557-1.095) | 0.152 |  | 0.648(0.443-0.946) | 0.025 |
|  | No | 1.000 |  |  | 1.000 |  |
| Eating speed | Fast | 0.825(0.615-1.106) | 0.198 |  | 1.076(0.784-1.476) | 0.652 |
|  | Slow | 1.000 |  |  | 1.000 |  |
| Egg (g/week) | ≥350 | 0.949(0.674-1.336) | 0.764 |  | 1.403(0.989-1.991) | 0.058 |
|  | <350 | 1.000 |  |  | 1.000 |  |
| Food left overnight | ≥1 | 0.903(0.645-1.263) | 0.551 |  | 0.838(0.589-1.191) | 0.324 |
| (times/week) | <1 | 1.000 |  |  | 1.000 |  |
| Freshwater fish (times/week) | ≥1 | 0.927(0.667-1.289) | 0.651 |  | 0.737(0.511-1.065) | 0.104 |
|  | <1 | 1.000 |  |  | 1.000 |  |
| Fried food (times/week) | ≥1 | 0.953(0.709-1.282) | 0.752 |  | 0.756(0.550-1.038) | 0.084 |
|  | <1 | 1.000 |  |  | 1.000 |  |
| Fruits (g/week) | ≥1000 | 1.001(0.744-1.348) | 0.995 |  | 0.906(0.656-1.252) | 0.551 |
|  | <1000 | 1.000 |  |  | 1.000 |  |
| Garlic (times/week) | ≥1 | 1.052(0.770-1.437) | 0.750 |  | 1.181(0.848-1.644) | 0.325 |
|  | <1 | 1.000 |  |  | 1.000 |  |
| Environmental factors |  | *ZNF331* methylation | |  | *WIF1* methylation | |
|  |  | OR^a^ (95% CI) | *P* |  | OR^a^ (95% CI) | *P* |
| Green vegetables (g/week) | ≥250 | 1.277(0.815-2.000) | 0.285 |  | 1.240(0.766-2.008) | 0.381 |
|  | <250 | 1.000 |  |  | 1.000 |  |
| Hot food | Yes | 0.810(0.603-1.088) | 0.161 |  | 1.004(0.732-1.379) | 0.978 |
|  | No | 1.000 |  |  | 1.000 |  |
| Marine product (times/week) | ≥1 | 0.889(0.562-1.405) | 0.614 |  | 1.024(0.618-1.698) | 0.926 |
|  | <1 | 1.000 |  |  | 1.000 |  |
| Pork (g/week) | ≥250 | 1.053(0.777-1.427) | 0.739 |  | 0.905(0.654-1.251) | 0.545 |
|  | <250 | 1.000 |  |  | 1.000 |  |
| Refrigerated food | Yes | 1.185(0.858-1.638) | 0.303 |  | 1.447(0.999-2.095) | 0.051 |
|  | No | 1.000 |  |  | 1.000 |  |
| Salted food | Yes | 0.906(0.674-1.218) | 0.514 |  | 0.822(0.596-1.135) | 0.234 |
|  | No | 1.000 |  |  | 1.000 |  |
| Tea | Yes | 1.124(0.813-1.553) | 0.479 |  | 0.846(0.596-1.201) | 0.350 |
|  | No | 1.000 |  |  | 1.000 |  |
| Water | River-water and well-water | 1.022(0.728-1.435) | 0.898 |  | 0.895(0.618-1.296) | 0.558 |
|  | Tap water and mineral-water | 1.000 |  |  | 1.000 |  |
| Dairy products (times/week) | ≥1 | 0.794(0.589-1.070) | 0.130 |  | 1.217(0.880-1.682) | 0.235 |
|  | <1 | 1.000 |  |  | 1.000 |  |
| *H. pylori* infection | Positive | 0.645(0.477-0.872) | 0.004 |  | 0.962(0.699-1.323) | 0.812 |
|  | Negative | 1.000 |  |  | 1.000 |  |
| Smoking | Yes | 1.047(0.778-1.408) | 0.763 |  | 0.861(0.627-1.182) | 0.355 |
|  | No | 1.000 |  |  | 1.000 |  |

CI, confidence interval; OR odds ratio.

^a^ Adjusted for propensity score of age, sex, BMI, occupation, monthly income and family history of GC.
