# Supplementary material for: Association of ZNF331 and WIF1 methylation in peripheral blood leukocytes with the risk and prognosis of gastric cancer
Source: BMC Cancer. 2021 May 15;21:551. doi: 10.1186/s12885-021-08199-4 (PMC8126111; doi:10.1186/s12885-021-08199-4)
Supplement: Supplementary file 9 — Additional file 9: Table S6. Effects of the combination between environmental factors and ZNF331 methylation status on GC risk in the whole population and internal validation datasets. [file 12885_2021_8199_MOESM9_ESM.docx]

**Table S6** Effects of the combination between environmental factors and *ZNF331* methylation status on GC risk in the whole population and internal validation datasets.

| Combinations | Whole population  (N=795) | |  | Internal validation population  (N=530) | |
| --- | --- | --- | --- | --- | --- |
|  | OR^a^ | 95%CI |  | OR^b^ | 95%CI^c^ |
| *ZNF331* methylation & Green vegetables |  |  |  |  |  |
| -/- | 1.000 |  |  | 1.000 |  |
| +/- | 0.188 | 0.058-0.603 |  | 0.189 | 0.058-0.399 |
| -/+ | 0.126 | 0.046-0.340 |  | 0.129 | 0.041-0.224 |
| +/+ | 0.073 | 0.027-0.196 |  | 0.077 | 0.024-0.131 |
| *ZNF331* methylation & Garlic |  |  |  |  |  |
| -/- | 1.000 |  |  | 1.000 |  |
| +/- | 0.576 | 0.383-0.868 |  | 0.589 | 0.435-0.768 |
| -/+ | 0.352 | 0.211-0.586 |  | 0.369 | 0.244-0.538 |
| +/+ | 0.138 | 0.080-0.238 |  | 0.150 | 0.100-0.215 |

CI, confidence interval; OR, odds ratio; GC, gastric cancer.

“+” represents *ZNF331* high methylation or high intakes of green vegetable (≥250 g/week) or garlic (≥1 times/week) depends on the combinations in order; “-” represents *ZNF331* low methylation or low intakes of green vegetable (<250 g/week) or garlic (<1 times/week) depends on the order of combinations.

^a^ OR adjusted for propensity score of age, sex, BMI, occupation, monthly income and family history of GC.

^b^ Median odds ratio was derived from the internal validation datasets generated by repeating 1000 times of subsampling without replacement.

^c^ Intervals from the 2.5th to the 97.5th percentile.
