# Supplementary material for: Association of ZNF331 and WIF1 methylation in peripheral blood leukocytes with the risk and prognosis of gastric cancer
Source: BMC Cancer. 2021 May 15;21:551. doi: 10.1186/s12885-021-08199-4 (PMC8126111; doi:10.1186/s12885-021-08199-4)
Supplement: Supplementary file 10 — Additional file 10: Table S7. Effects of the combination and interaction between environmental factors and ZNF331 methylation status on GC risk. [file 12885_2021_8199_MOESM10_ESM.docx]

**Table S7** Effects of the combination and interaction between environmental factors and *ZNF331* methylation status on GC risk

| Environmental factors |  | *ZNF331* methylation status | | | | | | | |
| --- | --- | --- | --- | --- | --- | --- | --- | --- | --- |
|  |  | Hm | |  | Lm | |  | Interactions | |
|  |  | OR^a^ (95% CI) | *P* |  | OR^a^ (95% CI) | *P* |  | OR^b^ (95% CI) | *P* |
| Alcohol consumption | Yes | 0.774(0.487-1.229) | 0.277 |  | 1.480(0.912-2.401) | 0.112 |  | 1.019(0.528-1.967) | 0.955 |
|  | No | 0.513(0.317-0.831) | 0.007 |  | 1.000 |  |  |  |  |
| Beef and mutton (g/week) | ≥250 | 0.229(0.115-0.456) | <0.001 |  | 0.527(0.275-1.007) | 0.053 |  | 0.815(0.318-2.087) | 0.669 |
|  | <250 | 0.534(0.375-0.762) | 0.001 |  | 1.000 |  |  |  |  |
| Irregular diet | Yes | 1.345(0.786-2.301) | 0.280 |  | 4.275(2.352-7.769) | <0.001 |  | 0.497(0.223-1.106) | 0.087 |
|  | No | 0.633(0.431-0.930) | 0.020 |  | 1.000 |  |  |  |  |
| Egg (g/week) | ≥350 | 0.273(0.157-0.475) | <0.001 |  | 0.615(0.356-1.063) | 0.081 |  | 0.819(0.379-1.767) | 0.611 |
|  | <350 | 0.542(0.370-0.793) | 0.002 |  | 1.000 |  |  |  |  |
| Food left overnight | ≥1 | 1.258(0.724-2.185) | 0.416 |  | 2.355(1.344-4.127) | 0.003 |  | 1.076(0.491-2.356) | 0.855 |
| (times/week) | <1 | 0.496(0.250-0.985) | 0.045 |  | 1.000 |  |  |  |  |
| Freshwater fish (times/week) | ≥1 | 2.451(1.436-4.184) | 0.001 |  | 3.435(1.929-6.117) | <0.001 |  | 1.578(0.715-3.483) | 0.258 |
|  | <1 | 0.452(0.304-0.672) | <0.001 |  | 1.000 |  |  |  |  |
| Fried food (times/week) | ≥1 | 0.770(0.482-1.230) | 0.275 |  | 1.924(1.188-3.117) | 0.008 |  | 0.601(0.312-1.157) | 0.127 |
|  | <1 | 0.666(0.425-1.044) | 0.076 |  | 1.000 |  |  |  |  |
| Refrigerated food | Yes | 0.193(0.112-0.333) | <0.001 |  | 0.353(0.203-0.612) | <0.001 |  | 1.163(0.551-2.454) | 0.692 |
|  | No | 0.471(0.250-0.887) | 0.020 |  | 1.000 |  |  |  |  |
| Salted food | Yes | 1.098(0.692-1.743) | 0.692 |  | 2.327(1.427-3.793) | 0.001 |  | 0.830(0.424-1.625) | 0.586 |
|  | No | 0.569(0.364-0.890) | 0.013 |  | 1.000 |  |  |  |  |
| Dairy products (times/week) | ≥1 | 0.954(0.594-1.531) | 0.845 |  | 1.756(1.089-2.831) | 0.021 |  | 1.025(0.530-1.981) | 0.941 |
|  | <1 | 0.530(0.329-0.853) | 0.009 |  | 1.000 |  |  |  |  |
| Water | River-water and well-water | 0.873(0.522-1.459) | 0.604 |  | 2.140(1.199-3.821) | 0.010 |  | 0.724(0.331-1.585) | 0.420 |
|  | Tap water and mineral-water | 0.563(0.385-0.824) | 0.003 |  | 1.000 |  |  |  |  |
| *H. pylori* infection | Positive | 1.009(0.609-1.672) | 0.971 |  | 1.632(0.983-2.709) | 0.058 |  | 1.289(0.644-2.580) | 0.472 |
|  | Negative | 0.480(0.287-0.803) | 0.005 |  | 1.000 |  |  |  |  |

Lm, low methylation; Hm, high methylation; CI, confidence interval; OR, odds ratio; GC, gastric cancer.

^a^ Combined effects adjusted for propensity score of age, sex, BMI, occupation, monthly income and family history of GC.

^b^ Interactions adjusted for propensity score of age, sex, BMI, occupation, monthly income and family history of GC.
