# Supplementary material for: Association of ZNF331 and WIF1 methylation in peripheral blood leukocytes with the risk and prognosis of gastric cancer
Source: BMC Cancer. 2021 May 15;21:551. doi: 10.1186/s12885-021-08199-4 (PMC8126111; doi:10.1186/s12885-021-08199-4)
Supplement: Supplementary file 11 — Additional file 11: Table S8. Effects of the combination and interaction between environmental factors and WIF1 methylation status on GC risk. [file 12885_2021_8199_MOESM11_ESM.docx]

**Table S8** Effects of the combination and interaction between environmental factors and *WIF1* methylation status on GC risk

| Environmental factors |  | *WIF1* methylation status | | | | | | | |
| --- | --- | --- | --- | --- | --- | --- | --- | --- | --- |
|  |  | Hm | |  | Lm | |  | Interactions | |
|  |  | OR^a^ (95% CI) | *P* |  | OR^a^ (95% CI) | *P* |  | OR^b^ (95% CI) | *P* |
| Alcohol consumption | Yes | 0.847(0.501-1.433) | 0.537 |  | 1.758(1.180-2.620) | 0.006 |  | 0.707(0.341-1.465) | 0.351 |
|  | No | 0.681(0.405-1.145) | 0.148 |  | 1.000 |  |  |  |  |
| Beef and mutton (g/week) | ≥250 | 0.484(0.207-1.133) | 0.095 |  | 0.397(0.226-0.696) | 0.001 |  | 2.535(0.883-7.274) | 0.084 |
|  | <250 | 0.482(0.326-0.710) | <0.001 |  | 1.000 |  |  |  |  |
| Irregular diet | Yes | 2.168(1.022-4.599) | 0.044 |  | 0.429(0.205-0.898) | 0.025 |  | 0.719(0.304-1.703) | 0.453 |
|  | No | 0.669(0.337-1.330) | 0.252 |  | 1.000 |  |  |  |  |
| Egg (g/week) | ≥350 | 0.416(0.230-0.753) | 0.004 |  | 0.544(0.346-0.857) | 0.009 |  | 1.504(0.677-3.339) | 0.316 |
|  | <350 | 0.509(0.332-0.779) | 0.002 |  | 1.000 |  |  |  |  |
| Food left overnight | ≥1 | 1.183(0.696-2.011) | 0.534 |  | 2.132(1.356-3.354) | 0.001 |  | 0.932(0.408-2.130) | 0.867 |
| (times/week) | <1 | 0.595(0.292-1.215) | 0.154 |  | 1.000 |  |  |  |  |
| Freshwater fish (times/week) | ≥1 | 2.901(1.469-5.731) | 0.002 |  | 4.229(2.658-6.729) | <0.001 |  | 1.254(0.518-3.038) | 0.616 |
|  | <1 | 0.547(0.352-0.850) | 0.007 |  | 1.000 |  |  |  |  |
| Fried food (times/week) | ≥1 | 0.945(0.560-1.595) | 0.833 |  | 1.456(0.984-2.154) | 0.060 |  | 1.306(0.633-2.693) | 0.470 |
|  | <1 | 0.497(0.300-0.825) | 0.007 |  | 1.000 |  |  |  |  |
| Garlic (times/week) | ≥1 | 0.183(0.099-0.340) | <0.001 |  | 0.283(0.183-0.436) | <0.001 |  | 1.248(0.560-2.782) | 0.589 |
|  | <1 | 0.520(0.333-0.814) | 0.004 |  | 1.000 |  |  |  |  |
| Green vegetables (g/week) | ≥250 | 0.152(0.077-0.298) | <0.001 |  | 0.260(0.139-0.486) | <0.001 |  | 1.220(0.372-4.004) | 0.743 |
|  | <250 | 0.478(0.156-1.470) | 0.198 |  | 1.000 |  |  |  |  |
| Refrigerated food | Yes | 0.227(0.136-0.380) | <0.001 |  | 0.306(0.195-0.479) | <0.001 |  | 2.588(1.097-6.105) | 0.030 |
|  | No | 0.287(0.136-0.604) | 0.001 |  | 1.000 |  |  |  |  |
| Salted food | Yes | 1.037(0.609-1.766) | 0.893 |  | 2.085(1.410-3.083) | <0.001 |  | 0.799(0.386-1.657) | 0.547 |
|  | No | 0.622(0.384-1.007) | 0.054 |  | 1.000 |  |  |  |  |
| Dairy products (times/week) | ≥1 | 1.080(0.666-1.752) | 0.754 |  | 1.465(0.993-2.161) | 0.054 |  | 2.135(1.004-4.540) | 0.049 |
|  | <1 | 0.345(0.193-0.619) | <0.001 |  | 1.000 |  |  |  |  |
| Water | River-water and well-water | 0.784(0.410-1.500) | 0.462 |  | 2.358(1.501-3.705) | <0.001 |  | 0.500(0.216-1.155) | 0.105 |
|  | Tap water and mineral-water | 0.665(0.437-1.013) | 0.057 |  | 1.000 |  |  |  |  |
| *H. pylori* infection | Positive | 1.116(0.675-1.847) | 0.669 |  | 1.882(1.254-2.827) | 0.002 |  | 1.198(0.560-2.563) | 0.641 |
|  | Negative | 0.495(0.279-0.878) | 0.016 |  | 1.000 |  |  |  |  |

Lm, low methylation; Hm, high methylation; CI, confidence interval; OR, odds ratio; GC, gastric cancer.

^a^ Combined effects adjusted for propensity score of age, sex, BMI, occupation, monthly income and family history of GC.

^b^ Interactions adjusted for propensity score of age, sex, BMI, occupation, monthly income and family history of GC.
