# Supplementary material for: Association of ZNF331 and WIF1 methylation in peripheral blood leukocytes with the risk and prognosis of gastric cancer
Source: BMC Cancer. 2021 May 15;21:551. doi: 10.1186/s12885-021-08199-4 (PMC8126111; doi:10.1186/s12885-021-08199-4)
Supplement: Supplementary file 12 — Additional file 12: Table S9. Effects of the combination and interaction between ZNF331 and WIF1 methylation on GC risk. [file 12885_2021_8199_MOESM12_ESM.docx]

**Table S9** Effects of the combination and interaction between *ZNF331* and *WIF1* methylation on GC risk

|  | | *WIF1* methylation status | | | | | | | | |
| --- | --- | --- | --- | --- | --- | --- | --- | --- | --- | --- |
|  |  | Hm |  |  | Lm |  |  | Interactions | |  |
|  |  | OR^a^ (95% CI) | *P* |  | OR^a^ (95% CI) | *P* |  | OR^b^ (95% CI) | *P* |  |
| *ZNF331* methylation status | Hm | 0.274(0.158-0.474) | <0.001 |  | 0.420(0.279-0.632) | <0.001 |  | 1.570(0.732-3.369) | 0.247 |  |
|  | Lm | 0.415(0.241-0.715) | 0.002 |  | 1.000 |  |  |  |  |  |

Lm, low methylation; Hm, high methylation; CI, confidence interval; OR, odds ratio; GC, gastric cancer.

^a^ Combined effects adjusted for propensity score of age, sex, BMI, occupation, monthly income and family history of GC.

^b^ Interactions adjusted for propensity score of age, sex, BMI, occupation, monthly income and family history of GC.
