# Supplementary material for: Association of ZNF331 and WIF1 methylation in peripheral blood leukocytes with the risk and prognosis of gastric cancer
Source: BMC Cancer. 2021 May 15;21:551. doi: 10.1186/s12885-021-08199-4 (PMC8126111; doi:10.1186/s12885-021-08199-4)
Supplement: Supplementary file 13 — Additional file 13: Table S10. Association between demographic characteristics and GC prognosis. [file 12885_2021_8199_MOESM13_ESM.docx]

**Table S10** Association between demographic characteristics and GC prognosis

| Demographic characteristics |  | Cases (%) | HR (95% CI) | *P* |
| --- | --- | --- | --- | --- |
| Sex | Male | 287(76.5) | 0.846(0.610-1.173) | 0.315 |
|  | Female | 88(23.5) | 1.000 |  |
| Age | ≥60 | 168(44.8) | 1.217(0.917-1.614) | 0.174 |
|  | <60 | 207(55.2) | 1.000 |  |
| BMI (kg/m^2^) | ≥24.00 | 119(31.7) | 0.889(0.650-1.215) | 0.459 |
|  | <24.00 | 256(68.3) | 1.000 |  |
| Monthly income (RMB/Per capita) | ≥1000 | 240(64.0) | 0.997(0.742-1.341) | 0.986 |
|  | <1000 | 135(36.0) | 1.000 |  |
| Occupation | White Collar | 137(36.5) | 1.158(0.864-1.553) | 0.326 |
|  | Blue Collar | 238(63.5) | 1.000 |  |
| Family history of gastric cancer | Yes | 49(13.1) | 1.126(0.744-1.702) | 0.575 |
|  | No | 326(86.9) | 1.000 |  |
| *H. pylori* infection | Positive | 238(63.5) | 0.933(0.692-1.260) | 0.652 |
|  | Negative | 137(36.5) | 1.000 |  |
| Gastric ulcer | Yes | 306(81.6) | 1.090(0.748-1.589) | 0.654 |
|  | No | 69(18.4) | 1.000 |  |
| Chronic gastritis | Yes | 316(84.3) | 1.255(0.812-1.941) | 0.306 |
|  | No | 59(15.7) | 1.000 |  |

CI, confidence interval; HR, hazard ratio; BMI, body mass index; GC, gastric cancer.
