# Supplementary material for: Association of ZNF331 and WIF1 methylation in peripheral blood leukocytes with the risk and prognosis of gastric cancer
Source: BMC Cancer. 2021 May 15;21:551. doi: 10.1186/s12885-021-08199-4 (PMC8126111; doi:10.1186/s12885-021-08199-4)
Supplement: Supplementary file 14 — Additional file 14: Table S11. Association between clinical characteristics and GC prognosis. [file 12885_2021_8199_MOESM14_ESM.docx]

**Table S11** Association between clinical characteristics and GC prognosis

| Clinical characteristics |  | Cases (%) | HR (95% CI) | *P* | HR^a^ (95% CI) | *P* |
| --- | --- | --- | --- | --- | --- | --- |
| Tumor site | Distal stomach | 231(61.6) | 0.758(0.563-1.020) | 0.068 | 0.760(0.565-1.023) | 0.070 |
|  | Others | 144(38.4) | 1.000 |  | 1.000 |  |
| Tumor size | ≥5cm | 178(47.5) | 1.858(1.379-2.505) | <0.001 | 1.878(1.390-2.538) | <0.001 |
|  | <5cm | 197(52.5) | 1.000 |  | 1.000 |  |
| Pathological type | Other type | 9(2.4) | 0.643(0.138-2.997) | 0.572 | 0.654(0.138-3.098) | 0.591 |
|  | Ulcer type | 60(16.0) | 0.713(0.344-1.478) | 0.362 | 0.728(0.349-1.516) | 0.394 |
|  | Infiltrating ulcer type | 214(57.1) | 1.183(0.652-2.145) | 0.579 | 1.198(0.661-2.170) | 0.551 |
|  | Infiltrating type | 60(16.0) | 1.740(0.902-3.359) | 0.098 | 1.756(0.910-3.390) | 0.093 |
|  | Polypoid type | 32(8.5) | 1.000 |  | 1.000 |  |
| Histological type | Adenocarcinoma | 200(53.3) | 1.200(0.783-1.838) | 0.401 | 1.193(0.778-1.829) | 0.417 |
|  | Particular types carcinoma | 109(29.1) | 0.884(0.548-1.427) | 0.614 | 0.900(0.557-1.454) | 0.667 |
|  | Mixed carcinoma | 66(17.6) | 1.000 |  | 1.000 |  |
| Differentiation | Middle to High | 149(39.7) | 0.753(0.546-1.038) | 0.083 | 0.749(0.541-1.036) | 0.081 |
|  | Low | 226(60.3) | 1.000 |  | 1.000 |  |
| TNM stage | Ⅳ | 217(57.9) | 5.972(2.681-13.301) | <0.001 | 5.886(2.639-13.131) | <0.001 |
|  | Ⅲ | 81(21.6) | 2.478(0.991-6.197) | 0.052 | 2.462(0.984-6.162) | 0.054 |
|  | Ⅱ | 21(5.6) | 1.319(0.388-4.482) | 0.657 | 1.299(0.382-4.421) | 0.675 |
|  | Ⅰ | 56(14.9) | 1.000 |  | 1.000 |  |
| CA19-9 | ≥37u/ml | 300(80.0) | 1.558(1.079-2.248) | 0.018 | 1.603(1.111-2.312) | 0.012 |
|  | <37u/ml | 75(20.0) | 1.000 |  | 1.000 |  |
| CEA | ≥5ng/ml | 295(78.7) | 1.562(1.116-2.187) | 0.009 | 1.577(1.122-2.217) | 0.009 |
|  | <5ng/ml | 80(21.3) | 1.000 |  | 1.000 |  |

CI, confidence interval; HR, hazard ratio; GC, gastric cancer.

^a^ Adjusted for age, sex, BMI.
