# Supplementary material for: Association of ZNF331 and WIF1 methylation in peripheral blood leukocytes with the risk and prognosis of gastric cancer
Source: BMC Cancer. 2021 May 15;21:551. doi: 10.1186/s12885-021-08199-4 (PMC8126111; doi:10.1186/s12885-021-08199-4)
Supplement: Supplementary file 15 — Additional file 15: Table S12. Multivariate analysis of GC prognosis. [file 12885_2021_8199_MOESM15_ESM.docx]

**Table S12** Multivariate analysis of GC prognosis

| Variable | | *β-coefficient* | SE | *P* | HR (95% CI) |
| --- | --- | --- | --- | --- | --- |
| Sex | Male | -0.095 | 0.170 | 0.577 | 0.909(0.651-1.269) |
|  | Female |  |  |  | 1.000 |
| Age | ≥60 | 0.165 | 0.145 | 0.257 | 1.179(0.887-1.568) |
|  | <60 |  |  |  | 1.000 |
| BMI (kg/m^2^) | ≥24.00 | -0.101 | 0.152 | 0.507 | 0.904(0.671-1.218) |
|  | <24.00 |  |  |  | 1.000 |
| Tumor size | ≥5cm | 0.417 | 0.148 | 0.005 | 1.518(1.137-2.027) |
|  | <5cm |  |  |  | 1.000 |
| TNM stage | Ⅳ | 1.728 | 0.346 | <0.001 | 5.629(2.855-11.102) |
|  | Ⅲ | 0.924 | 0.379 | 0.015 | 2.518(1.197-5.297) |
|  | Ⅱ | 0.343 | 0.558 | 0.539 | 1.409(0.472-4.205) |
|  | Ⅰ |  |  |  | 1.000 |

CI, confidence interval; HR, hazard ratio; GC, gastric cancer; SE, standard error.
