# Supplementary material for: Association of ZNF331 and WIF1 methylation in peripheral blood leukocytes with the risk and prognosis of gastric cancer
Source: BMC Cancer. 2021 May 15;21:551. doi: 10.1186/s12885-021-08199-4 (PMC8126111; doi:10.1186/s12885-021-08199-4)
Supplement: Supplementary file 16 — Additional file 16: Figure S4. Survival curves of the association between ZNF331 (a) and WIF1 (b) methylation and GC prognosis. [file 12885_2021_8199_MOESM16_ESM.docx]

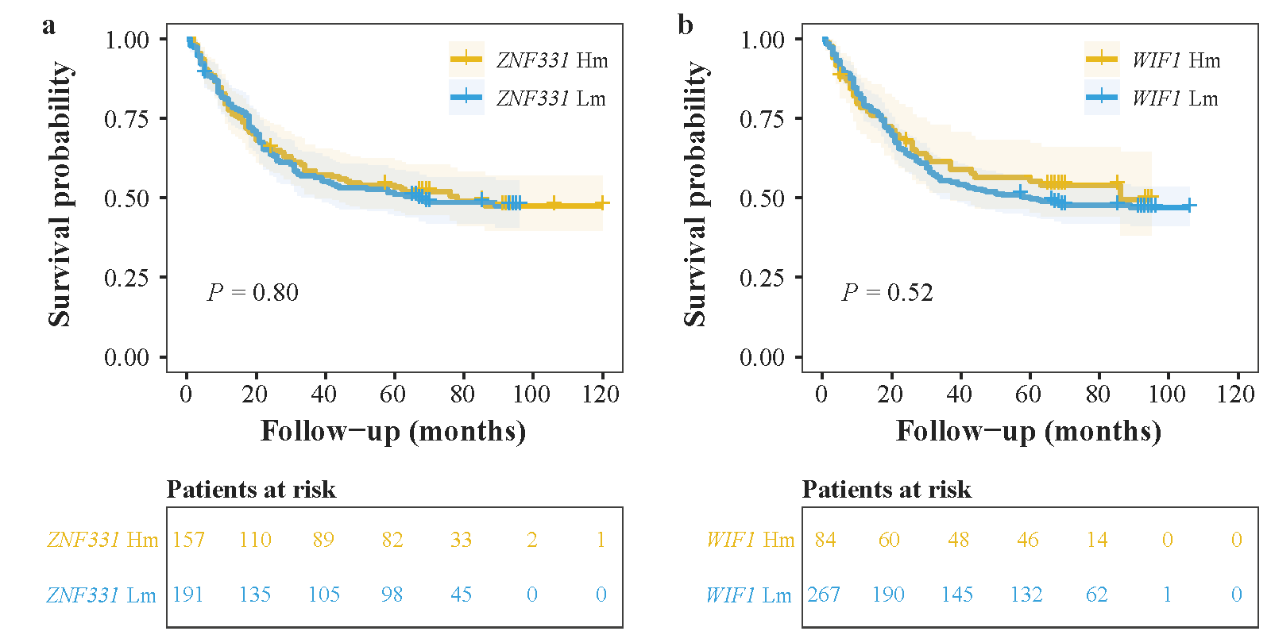


**Figure S4.** Survival curves of the association between *ZNF331* **(a)** and *WIF1* **(b)** methylation and GC prognosis. Hm, high methylation; Lm, low methylation.
