# Supplementary material for: Association of ZNF331 and WIF1 methylation in peripheral blood leukocytes with the risk and prognosis of gastric cancer
Source: BMC Cancer. 2021 May 15;21:551. doi: 10.1186/s12885-021-08199-4 (PMC8126111; doi:10.1186/s12885-021-08199-4)
Supplement: Supplementary file 17 — Additional file 17: Table S13. Association between the methylation status of genes and GC prognosis by stratified analysis. [file 12885_2021_8199_MOESM17_ESM.docx]

**Table S13** Association between the methylation status of genes and GC prognosis by stratified analysis

| Gene | | | | Male | | | | | | |  | Female | | | | | | |
| --- | --- | --- | --- | --- | --- | --- | --- | --- | --- | --- | --- | --- | --- | --- | --- | --- | --- | --- |
|  |  |  |  | Case (%) | HR^a^ (95% CI) | *P* | | HR^b^ (95% CI) | | *P* |  | Case (%) | HR^a^ (95% CI) | *P* | HR^b^ (95% CI) | | *P* | |
| *ZNF331* | | Hm | | 123(46.9) | 1.000(0.698-1.433) | 0.999 | | 0.937(0.635-1.382) | | 0.741 |  | 34(39.5) | 1.270(0.683-2.360) | 0.451 | 1.547(0.752-3.185) | | 0.236 | |
|  | | Lm | | 139(53.1) | 1.000 |  | | 1.000 | |  |  | 52(60.5) | 1.000 |  | 1.000 | |  | |
| *WIF1* | | Hm | | 63(23.3) | 0.643(0.411-1.008) | 0.054 | | 0.681(0.424-1.095) | | 0.113 |  | 21(25.9) | 1.878(0.952-3.705) | 0.069 | 1.288(0.540-3.076) | | 0.568 | |
|  | | Lm | | 207(76.7) | 1.000 |  | | 1.000 | |  |  | 60(74.1) | 1.000 |  | 1.000 | |  | |
| Gene | | | | <60 years | | | | | | |  | ≥60 years | | | | | | |
|  |  |  |  | Case (%) | HR^a^ (95% CI) | | *P* | | HR^b^ (95% CI) | *P* |  | Case (%) | HR^a^ (95% CI) | *P* | | HR^b^ (95% CI) | *P* | |
| *ZNF331* | | Hm | | 83(43.2) | 1.072(0.687-1.673) | | 0.758 | | 1.049(0.649-1.695) | 0.846 |  | 74(47.4) | 1.119(0.718-1.742) | 0.620 | | 1.083(0.675-1.738) | 0.741 | |
|  | | Lm | | 109(56.8) | 1.000 | |  | | 1.000 |  |  | 82(52.6) | 1.000 |  | | 1.000 |  | |
| *WIF1* | | Hm | | 43(21.9) | 0.637(0.361-1.124) | | 0.119 | | 0.622(0.351-1.103) | 0.104 |  | 41(26.5) | 1.015(0.612-1.685) | 0.953 | | 1.063(0.597-1.892) | 0.836 | |
|  | | Lm | | 153(78.1) | 1.000 | |  | | 1.000 |  |  | 114(73.5) | 1.000 |  | | 1.000 |  | |
| Gene | | | *H. Pylori* Negative | | | | | | |  | *H. Pylori* Positive | | | | | |  |  |
|  |  |  | Case (%) | HR^a^ (95% CI) | | *P* | | HR^b^ (95% CI) | *P* |  | Case (%) | HR^a^ (95% CI) | *P* | | HR^b^ (95% CI) | *P* |  |  |
| *ZNF331* | | Hm | | 62(49.6) | 1.456(0.869-2.438) | | 0.153 | | 1.563(0.896-2.727) | 0.115 |  | 95(42.6) | 0.915(0.610-1.373) | 0.667 | | 0.870(0.567-1.335) | 0.523 | |
|  | | Lm | | 63(50.4) | 1.000 | |  | | 1.000 |  |  | 128(57.4) | 1.000 |  | | 1.000 |  | |
| *WIF1* | | Hm | | 28(22.0) | 0.558(0.276-1.126) | | 0.103 | | 0.470(0.177-1.247) | 0.128 |  | 56(25.0) | 0.991(0.636-1.545) | 0.969 | | 0.939(0.584-1.509) | 0.795 | |
|  | | Lm | | 99(78.0) | 1.000 | |  | | 1.000 |  |  | 168(75.0) | 1.000 |  | | 1.000 |  | |
| Gene | | | TNM (Ⅰ+Ⅱ) | | | | | | |  | TNM (Ⅲ+Ⅳ) | | | | | |  |  |
|  |  |  | Case (%) | HR^a^ (95% CI) | | *P* | | HR^b^ (95% CI) | *P* |  | Case (%) | HR^a^ (95% CI) | *P* | | HR^b^ (95% CI) | *P* |  |  |
| *ZNF331* | | | Hm | 35(47.9) | 0.661(0.195-2.241) | | 0.505 | | 0.820(0.187-3.591) | 0.791 |  | 122(44.4) | 1.068(0.773-1.476) | 0.690 | | 1.044(0.745-1.464) | 0.801 | |
|  | | | Lm | 38(52.1) | 1.000 | |  | | 1.000 |  |  | 153(55.6) | 1.000 |  | | 1.000 |  | |
| *WIF1* | | | Hm | 21(29.6) | 1.130(0.304-4.198) | | 0.854 | | 0.997(0.213-4.676) | 0.997 |  | 63(22.5) | 0.934(0.635-1.375) | 0.730 | | 0.854(0.572-1.275) | 0.440 | |
|  | | | Lm | 50(70.4) | 1.000 | |  | | 1.000 |  |  | 217(77.5) | 1.000 |  | | 1.000 |  | |
| Gene | | | <5cm | | | | | | |  | ≥5cm | | | | | |  |  |
|  |  |  | Case (%) | HR^a^ (95% CI) | | *P* | | HR^b^ (95% CI) | *P* |  | Case (%) | HR^a^ (95% CI) | *P* | | HR^b^ (95% CI) | *P* |  |  |
| *ZNF331* | | | Hm | 79(43.9) | 0.856(0.509-1.438) | | 0.555 | | 0.879(0.506-1.527) | 0.645 |  | 78(46.4) | 1.312(0.844-2.041) | 0.227 | | 1.271(0.806-2.004) | 0.301 | |
|  | | | Lm | 101(56.1) | 1.000 | |  | | 1.000 |  |  | 90(53.6) | 1.000 |  | | 1.000 |  | |
| *WIF1* | | | Hm | 41(22.7) | 0.740(0.408-1.344) | | 0.323 | | 0.680(0.362-1.275) | 0.229 |  | 43(25.3) | 0.895(0.543-1.476) | 0.664 | | 0.828(0.476-1.439) | 0.502 | |
|  | | | Lm | 140(77.3) | 1.000 | |  | | 1.000 |  |  | 127(74.7) | 1.000 |  | | 1.000 |  | |

Lm, low methylation; Hm, high methylation; CI, confidence interval; HR, hazard ratio; GC, gastric cancer.

^a^ Adjusted for age, sex, BMI, tumor size and TNM stage except stratified factors.

^b^ Adjusted for propensity score of all variables except stratified factors.
